# Efficacy of plasma exchange for antineutrophil cytoplasmic antibody-associated systemic vasculitis: a systematic review and meta-analysis

**DOI:** 10.1186/s13075-021-02415-z

**Published:** 2021-01-14

**Authors:** Yosuke Yamada, Makoto Harada, Yuuta Hara, Ryohei Iwabuchi, Koji Hashimoto, Shuhei Yamamoto, Yuji Kamijo

**Affiliations:** 1 Department of Nephrology, Shinshu University School of Medicine, 3-1-1 Asahi, Matsumoto, Nagano, 390-8621 Japan; 2 Department of Rehabilitation, Shinshu University Hospital, 3-1-1 Asahi, Matsumoto, Nagano, 390-8621 Japan

**Keywords:** Microscopic polyangiitis, Churg-Strauss syndrome, Granulomatosis with polyangiitis, Plasma exchange, Meta-analysis

## Abstract

**Objective:**

To assess through systematic review and meta-analysis whether plasma exchange (PE) is associated with prognosis in antineutrophil cytoplasmic antibody (ANCA)-associated vasculitis (AAV) patients.

**Methods:**

A systematic search of PubMed, MEDLINE, Embase, and CENTRAL databases from inception to 17 June 2020 was conducted. Ongoing or unpublished trials were also searched in ClinicalTrials.gov and the World Health Organization trials portal. Randomised controlled trials (RCTs) comparing PE vs. non-PE in AAV patients (microscopic polyangiitis [MPA], granulomatosis with polyangiitis [GPA], or eosinophilic granulomatosis with polyangiitis [EGPA]) were included. The combined risk ratio (RR) was calculated by the random-effects model using the Mantel-Haenszel method. Heterogeneity was measured using the *I*^2^ statistic. Primary outcomes were mortality, clinical remission (CR), and adverse events (AEs).

**Results:**

Four RCTs comparing PE vs. no PE (*N* = 827) and 1 RCT comparing PE vs. pulse steroid treatment (*N* = 137) were included. All participants were MPA or GPA patients (no EGPA patients). PE was not associated with main primary outcomes compared with no PE (mortality RR 0.93 [95% confidence interval {CI} 0.70–1.24], *I*^2^ = 0%; CR RR 1.02 [95% CI 0.91–1.15], *I*^2^ = 0%; and AE RR 1.10 [95% CI 0.73–1.68], *I*^2^ = 37%) or pulse steroid (mortality RR 0.99 [95% CI 0.71–1.37]; CR [the Birmingham Vasculitis Activity score] mean difference − 0.53 [95% CI − 1.40–0.34]; and AE RR 1.05 [95% CI 0.74–1.48]). Focusing on the early treatment phases, PE was associated with a reduction in end-stage renal disease incidence compared with both no PE (PE 1/43 vs. no PE 10/41; RR 0.14 [0.03–0.77] at 3 months) and pulse steroid (PE 11/70 vs. pulse steroid 23/67; RR 0.46 [0.24–0.86] at 3 months).

**Conclusion:**

We carried out a systematic review and meta-analysis targeting all AAV patients, including MPA, GPA, and EGPA. In AAV patients, performing PE was not associated with the risk of mortality, CR, and AE. No RCT exists evaluating the efficacy of PE for EGPA; hence, this is required in the future. The results may affect the development of guidelines for AAV and may indicate the direction of future clinical research on AAV.

**Trial registration:**

UMIN R000045239, PROSPERO CRD42020182566.

**Supplementary Information:**

The online version contains supplementary material available at 10.1186/s13075-021-02415-z.

## Background

Antineutrophil cytoplasmic antibody (ANCA)-associated vasculitis (AAV) is a systemic inflammatory condition characterised by ANCA production and serum positivity that injures small- to medium-sized blood vessels in body organs [1]. AAV conditions include microscopic polyangiitis (MPA), granulomatosis with polyangiitis (GPA), and eosinophilic granulomatosis with polyangiitis (EGPA) [2–6]. Although AAV with only renal lesions is called renal-limited vasculitis (RLV), RLV is usually interpreted as an organ-limited variant of MPA [7–9]. AAV often presents not only as rapidly progressive glomerulonephritis in the kidneys but also as interstitial pneumonia, alveolar haemorrhage, cranial nerve lesion, and others [2–5, 7, 8], with some of these being life-threatening. Therefore, immediate confirmation of the diagnosis and initiation of effective induction therapy are needed.

AAV is caused by autoimmune mechanisms and its treatment typically includes immunosuppressive agents (such as cyclophosphamide and rituximab) and combined glucocorticoid therapy for induction immunosuppressive therapy [10]. The main ANCA target antigens are myeloperoxidase and proteinase 3 [11]. Recently, ANCAs have been reported to activate neutrophils directly, which then adhere to and penetrate the vessel walls [12]. The activated neutrophils release various inflammatory mediators and factors that stimulate the alternative complement pathway [13], as well as neutrophil extracellular trap (NETs) formation [14].

Thus, as ANCAs appear to be associated with the progression of severe vasculitis lesions, eliminating ANCAs and their various mediators by plasma exchange (PE) may be an effective addition to immunosuppressive therapy for AAV patients [15]. However, since PE replaces a large amount of the patient’s plasma with almost the same amount of albumin preparation or fresh frozen plasma, side effects such as hypocalcaemia, hypo- or hypervolaemia, and anaphylactoid reactions are reported [16].

Several randomised controlled trials (RCTs) have investigated the efficacy and safety of PE for AAV. Moreover, the results of the largest RCT, the PEXIVAS study, were recently published [17]. Further, some systematic reviews of such clinical questions have been conducted previously [18–20]. However, since most of their study search occurred over 5 years ago, the results of recent studies, such as the PEXIVAS study, were not included [18, 19]. Moreover, the latest review’s search strategy did not include all three AAV subtypes, and the eligibility criteria might have excluded AAV without renal lesions [20]. AAV is dealt with as one disease entity in many guidelines, regardless of its subtype or lesion location [3–5]; hence, a review subsuming all the AAV types is more useful for guideline developers and clinicians.

Here, we performed a systematic review and meta-analysis of RCTs, with a search scope, including MPA, GPA, and EGPA, to assess the efficacy of PE for AAV. The primary outcomes were mortality, clinical remission (CR), and adverse events (AEs).

## Methods

The present systematic review and meta-analysis were performed in accordance with the Cochrane Handbook for Systematic reviews and Interventions. This study was reported following the Preferred Reporting Items for Systematic Reviews and Meta-Analysis (PRISMA) statement for health care interventions as shown in the additional PRISMA file (Additional file 1). The detailed method of this review is in the study protocol as shown in the additional data file (Additional file 2).

### Search strategy

We searched MEDLINE, PubMed, Embase, CENTRAL (Cochrane Central Register of Controlled Trials) databases to identify the studies. The final search date was 17 June 2020. The search strategies used with each database were created under the guidance of a Cochrane information specialist and are described in Additional file 3. We searched ClinicalTrials.gov and the World Health Organization trials portal to identify ongoing or unpublished trials.

### Study selection

Two reviewers (YY and MH) independently screened for RCTs comparing PE with non-PE in AAV patients. Only RCTs that met the following criteria were included in this review.

### Participants

Inclusion criteria included all studies that are primarily on AAV in participants aged ≥ 18 years who are diagnosed with AAV, including confirmed GPA (formerly Wegener’s granulomatosis; WG), EGPA (formerly Churg-Strauss syndrome; CSS), MPA, and RLV [6].

Exclusion criteria were patients with other types of vasculitis, including anti-glomerular basement membrane disease.

### Intervention and comparator

This review considered studies that evaluated the effectiveness of PE in AAV.

### Intervention: PE group

#### Comparator: non-PE or sham PE group

We included any method of PE treatment or PE dose. In reference to the American Society for Apheresis guidelines, PE was defined as typical PE, double filtration plasmapheresis, or selective PE [21–23]. Immunoabsorption treatment was excluded [24].

We included studies whose design could be used to evaluate the effect of PE alone, i.e. PE + treatment A compared with non-PE (or sham PE) + treatment A. If PE + treatment A was compared with non-PE (or sham PE) + no other treatment or if PE was compared directly with another treatment, we assessed those results separately.

In the case of a study with a mixture of eligible and ineligible participants, it was included if > 80% of all the participants were considered eligible.

### Data abstraction

Information regarding the study design, detailed content of intervention and comparator, number of participants, inclusion and exclusion criteria, and clinical outcomes were obtained. Missing data were requested from the corresponding author via email. The quality of the abstracted studies was assessed using Cochrane Collaboration’s tool for assessing the risk of bias (the Cochrane ‘Risk of bias’ tool 2) [25]. For the assessment of reporting bias, we planned to check using funnel plots if 10 or more studies were included; however, since the studies were not up to 10, the assessment was not performed.

### Outcomes

The primary outcomes were (1) mortality, (2) CR (as defined by the study’s authors, typically as the complete absence of disease activity determined by the Birmingham Vasculitis Activity Score [BVAS]), and (3) AEs. The secondary outcomes were (1) renal failure (end-stage renal disease [ESRD], the composite of ESRD or death, improvement in renal function or changes in serum creatinine level); (2) disease flare/relapse (as defined in the study); (3) health-related quality of life (QOL); and (4) disease damage according to the Vasculitis Damage Index (VDI).

### Statistical analysis

Data were analysed from June to July 2020. All the analyses were carried out using Review Manager 5, Version 5.3 (Copenhagen, Nordic Cochrane Centre, Germany). Dichotomous data were analysed using risk ratios (RR) with 95% confidence intervals (CIs). Continuous data were analysed as mean differences with 95% CIs when the measurements used the same scale. The pooled RR was calculated by the random-effect model using the Mantel-Haenszel method. For the assessment of statistical heterogeneity, we utilised the *I*^2^ statistic. Significant heterogeneity was defined as *I*^2^ statistics value of above 50%. Two-sided *P* < 0.05 was considered significant and was calculated using the *z* test of the null hypothesis indicating that there was no average effect in the random-effect model of PE vs. non-PE.

### Subgroup analysis and sensitivity analysis

We undertook the following subgroup analyses in the studies that had the following available data: types of AAV (only MPA or GPA) and PE conditions, while those of ANCA status and localisation of lesions could not be performed.

We performed sensitivity analyses on primary outcomes to determine if a high risk of bias that occurred in some included studies affected the study results by the exclusion of studies at a high risk of bias, exclusion of trials with ≤ 10 events, and exclusion of cluster RCTs, and by comparing fixed- vs. random-effects pooled estimates.

## Results

### The search and selection of studies

**Fig. 1:**
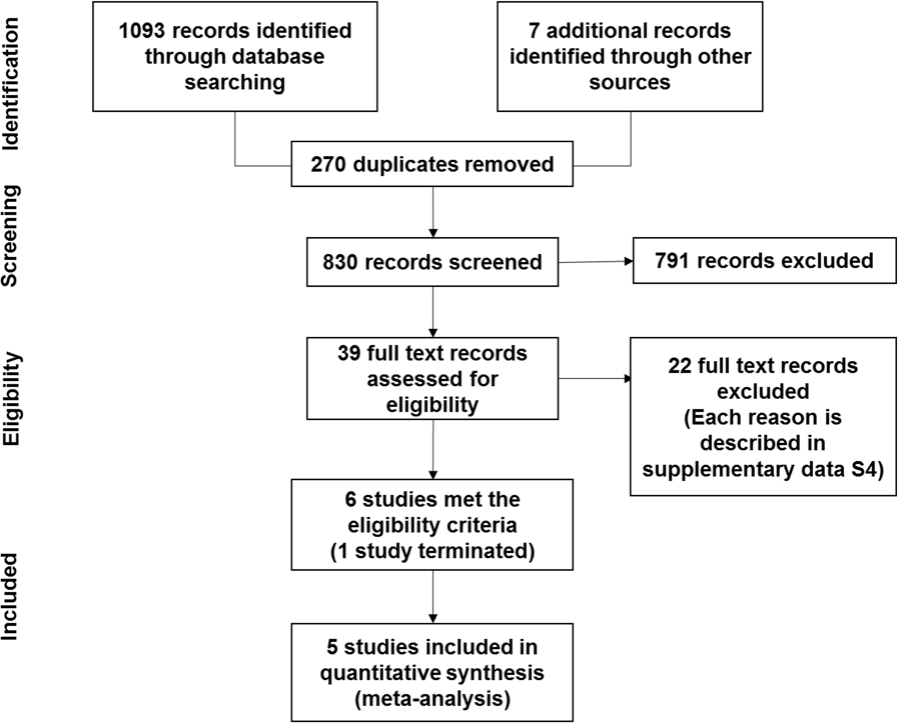
PRISMA flow diagram of this review

Figure 1 shows the PRISMA flow chart for the study selection. In total, 830 abstracts and titles were identified, and 39 were selected for full-text or abstract (unpublished) review. Twenty-two full-text records were excluded (the reasons are described in Additional file 4). Therefore, six RCTs fulfilled the eligibility criteria [17, 26–31]. Given that one of these studies, which was terminated before enrolling the target number of patients, did not have data [29], five studies were finally included in the quantitative synthesis. Four trials [17, 28, 30, 31] were on PE vs. no PE, while one trial [26, 27] was on PE vs. pulse steroid treatment [32].

### PE vs. no PE

#### Included studies

Four RCTs on PE vs. no PE involving 827 AAV patients (weighted mean age, 61 years, 42% female) were included. Table 1 summarises the characteristics of the included studies. All the patients included in this review either had MPA or GPA, and none had EGPA. Albumin substitution was the prevalent PE method. The maximum observational period was 5–11 (median or mean 2.0–4.6 years) years. The supplementary summary of included RCTs in this review is shown in Supplementary Table S1 (Additional file 5). Three papers reported C-ANCA positive rates (min-max: 41–100%), and 2 papers reported P-ANCA positive rates (18–59%). The prevalence of renal lesions (69–100%) was reported in all articles. The prevalence of lung lesions (42% and 66%) was reported in 2 papers. Glucocorticoid and cyclophosphamide combined treatments were used in all studies, but the presence or absence of other treatments varied.

**Table 1:** Summary of included PE vs no PE RCTs in this review

Source	Inclusion criteria	Exclusion criteria	No. of patients	Interventions	Primary outcome
PEXIVAS 2020 [17]	New or relapsing GPA or MPA; PR3 or MPO-ANCA positive; renal or pulmonary involvement	Age < 15 year; pregnancy; vasculitis other than MPA or GPA; anti-GBM disease; dialysis for greater than 21 days prior to randomisation or prior renal transplant; prior PE in 3 months; use of CYC, rituximab, or high dose GC prior to randomisation*	704, MPA or GPA (no data of percentage)	PE^†^ [(a) centrifugation or filter separation, (b) 3–5% albumin or fresh frozen plasma, (c) 60 mL/kg, (d) 7 sessions over 14 days] vs no PE	The composite of the death or ESRD
Szpirt et al., 2011 [28]	At least 2 of the following 3 criteria (i) WG-clinical manifestations at least 2 organs, (ii) histology-proven WG, (iii) positive ‘C-ANCA/PR3-ANCA’	No description	32 GPA (100%)	PE^†^ [(a) filter separation, (b) 3% albumin in Ringer’s lactate, (c) 4 L, (d) 6 sessions every other day. If high ANCA titre after 6 sessions, 3–6 sessions were added.] vs no PE	Renal progression, ESRD, improvement of renal function, remission, relapse, death
Zäuner et al., 2002 [31]	The clinical picture of type II or III RPGN [33]; Had not treated previously with immunosuppression or PE.	Type I RPGN [33]	39, MPA (18%), GPA (67%) or type II RPGN (15%)	PE^†^ [(a) no description, (b) fresh frozen plasma, (c) 40 mL/kg, (d) the mean 6 sessions (range, 3–12).] vs no PE	The composite of the death or ESRD, renal function, extrarenal manifestation, adverse events
Pusey et al., 1991 [30]	Impaired renal function; Focal necrotizing glomerulonephritis with crescents; a diagnosis of WG, MPA or IRPGN	Concomitant vasculitis other than AAV; anti-GBM disease; underlying chronic glomerulonephritis; previously treated with intravenous GC, oral CYC or PE*	52, MPA (42%), GPA (48%) or IRPGN (10%)^‡^	PE^†^ [(a) centrifugation, (b) 5% albumin, (c) 4 L, (d) 5 times within the first week. Mean 9 sessions (range 5–25).] vs no PE	ESRD, death, serum creatinine, improvement of renal function

*RCT* randomised controlled trial, *PE* plasma exchange; *GPA* granulomatosis with polyangiitis, *MPA* microscopic polyangiitis, *GBM* glomerular basement membrane, *CYC* cyclophosphamide, *GC* glucocorticoid, *ESRD* end-stage renal disease, *WG* Wegener’s granulomatosis, *RPGN* rapidly progressive glomerulonephritis, *IRPGN* idiopathic RPGN

*Partially omitted

^†^The detailed methods of PE are described in parentheses. (a) Separation method, (b) replacement fluid, (c) dose per session, and (d) number of sessions

^‡^Since 4 of 52 did not have data about the type, they are the ratios in 48 patients

#### Clinical outcomes

**Fig. 2:**
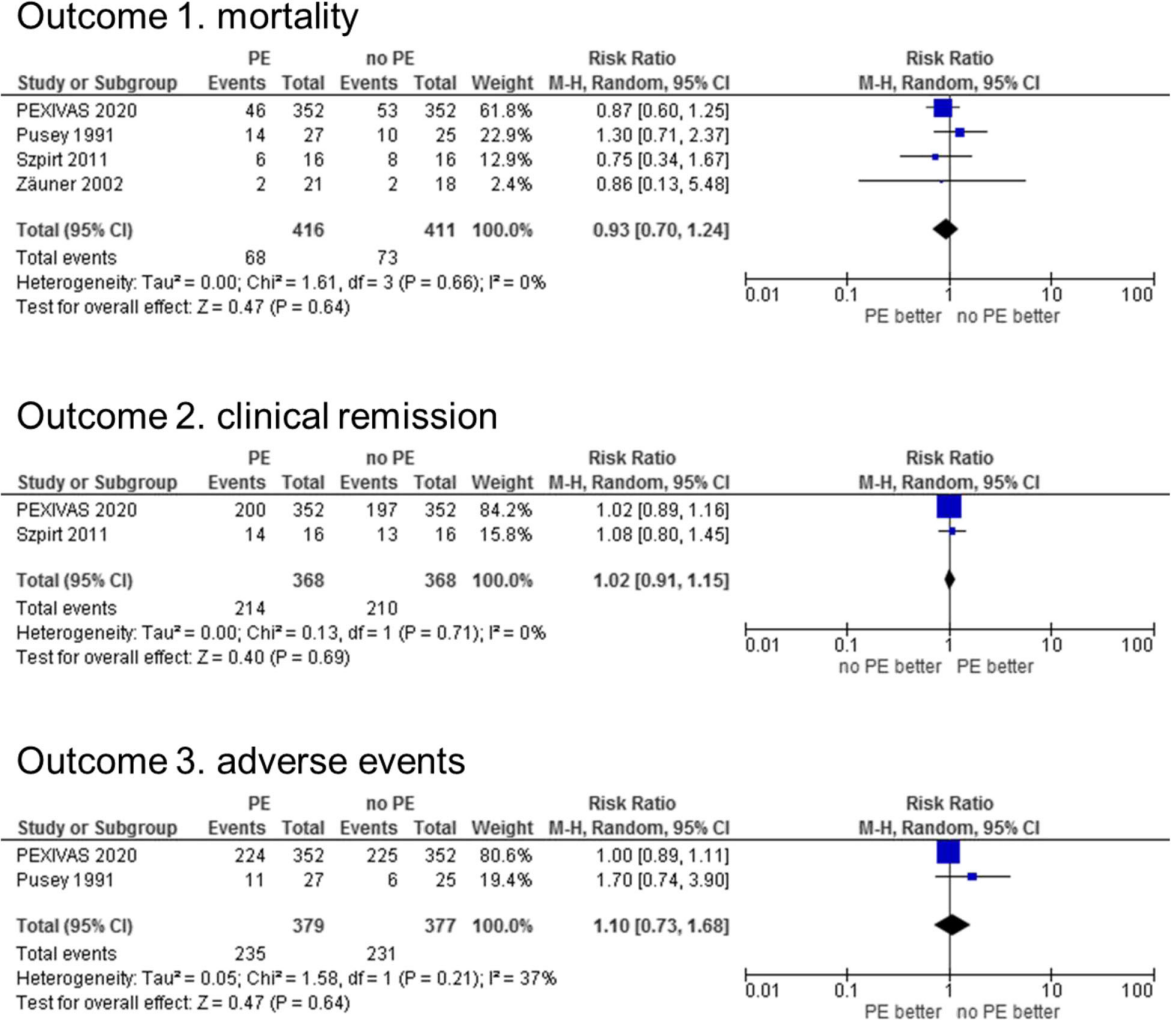
Forrest plots of primary outcomes between the PE and no PE groups. Regarding the timing of outcome measurement in this figure, mortality and adverse events are for the overall observational period, and clinical remission is at 1 year after the allocation. See Additional file 5 for other data. Abbreviation: PE, plasma exchange

All four studies reported on mortality. There was no statistically significant difference in the number of deaths throughout the overall observational period: 68 (16%) and 73 (18%) deaths occurred in the PE and no PE groups, respectively (RR 0.93 [95% CI, 0.70–1.24], *I*^2^ = 0%) (Fig. 2). Mortality RR was 0.54 (95% CI, 0.21–1.38), 0.71 (95% CI 0.27–1.86), 1.00 (95% CI 0.60–1.68), and 0.86 (95% CI 0.13–5.48) after 6 months, 1, 5, and 10 years, respectively. The detailed forest plot of mortality in patients with PE or no PE is shown in Supplementary figure S1 (Additional file 5).

Two studies reported CR (Fig. 2). The definition of remission varied depending on the study, and one paper defined that the BVAS for GPA condition = 0 was achieved and maintained [17]. Another paper defined this as the reduction in creatinine level by > 15% from that at study inclusion [28]. Since CR is a reversible outcome, CR at 1 year after allocation was used as a representative value. There was no statistically significant difference in the number of patients in remission at 1 year after allocation: 214 (58%) and 210 (57%) patients in the PE and no PE groups, respectively (RR 1.02 [95% CI, 0.91–1.15], *I*^2^ = 0%). The CR RR was 1.57 (95% CI, 1.07–2.30), 1.67 (95% CI, 1.06–2.61), and 2.20 (95% CI, 0.99–4.89) after 1 month, 3 months, and 5 years, respectively. There were statistically significant associations between PE and CR early after treatment. Supplementary figure S2 shows the detailed forest plot of the clinical remission of patients with and without PE (Additional file 5).

Two studies reported AEs (Fig. 2). According to Zäuner et al., no significant difference was observed in the incidence of possible side effects between the two treatment groups, with no detailed data [31]. A total of 235 (62%) and 231 (61%) AEs occurred in the PE and no PE groups, respectively, (RR 1.10 [95% CI, 0.73–1.68], *I*^2^ = 37%), with no significant difference. The RRs were 1.29 (95% CI 0.94–1.76), 1.20 (95% CI 0.98–1.46), 3.00 (95% CI 0.82–10.99), 0.87 (95% CI 0.56–1.35), 1.14 (95% CI 0.75–1.74), and 0.96 (95% CI 0.57–1.63) for cardiovascular disease, infections, and endocrine, gastrointestinal, kidney/urinary, and haematologic diseases, respectively. Supplementary figure S3 shows the detailed forest plot of AEs in patients with and without PE (Additional file 5).

**Fig. 3:**
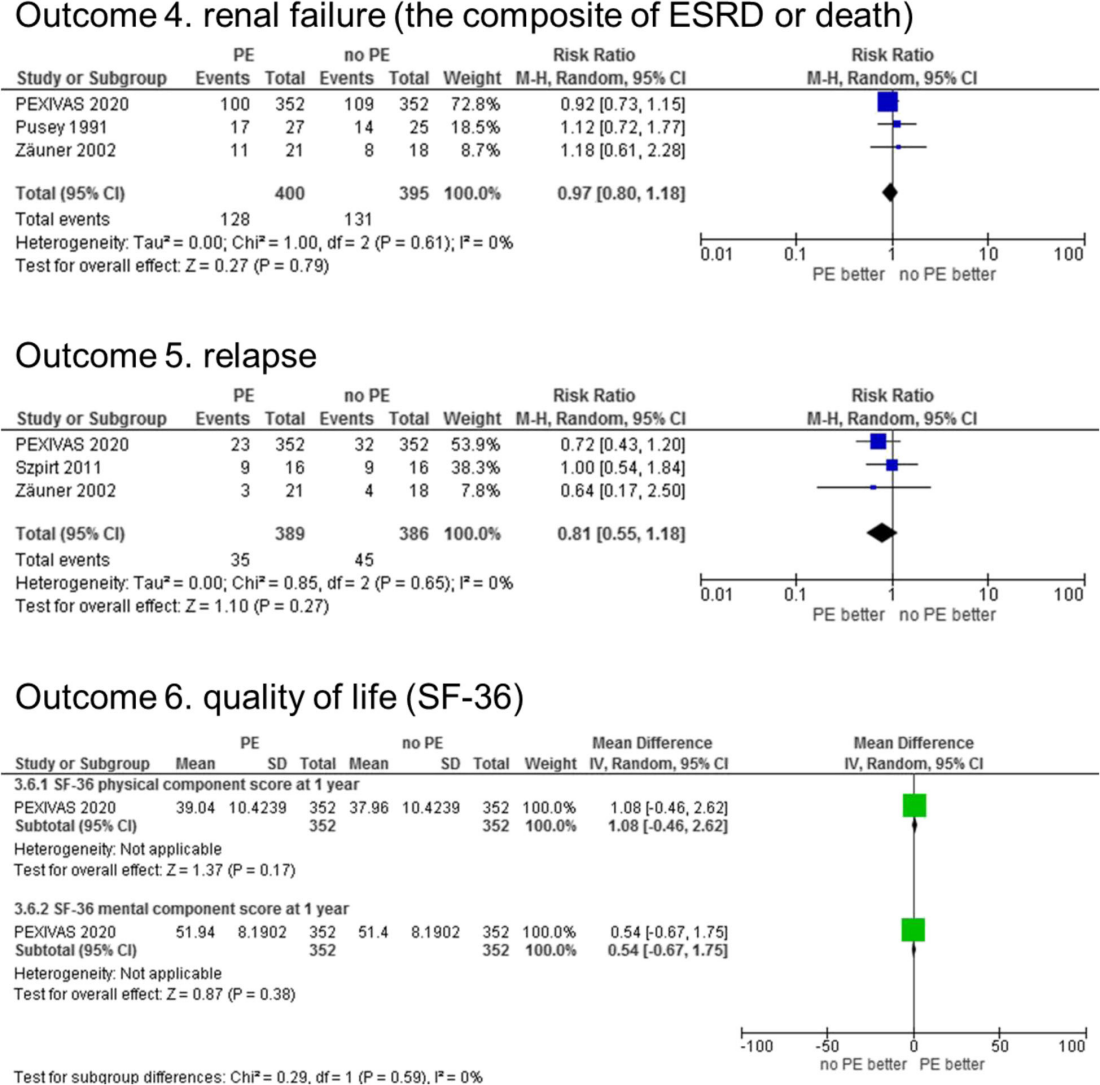
Forrest plots of the secondary outcomes between the PE and no PE groups. The composite of ESRD or death is shown as a representative of outcomes on renal failure, and SF-36 is shown as a representative of the quality of life indicators. Regarding the timing of outcome measurement, renal failure and relapse are for the overall observational period, and quality of life was at 1 year after the allocation. See Additional file 5 for other data. Abbreviations: PE, plasma exchange; ESRD, end-stage renal disease

Regarding renal failure (secondary outcome), the composite ESRD or death RR was 0.97 (95% CI 0.80–1.18), *I*^2^ = 0% for the overall observational period in the PE group as compared to the no PE group (Fig. 3). Refer to details in Supplementary figure S4 (Additional file 5) for the results by outcome measurement timing. For ESRD (death-censored), the RR was 0.85 (95% CI 0.57–1.28), *I*^2^ = 27% during the overall observational period. By the timing of outcome measurement, the risk was significantly lower in the PE group in the early post-treatment period. One (2%) and 10 (24%) cases of ESRD were observed in PE and no PE groups, respectively at 1 month and 3 months. The RRs tend to be lower when the post-treatment period was earlier (1-month RR 0.14 [95% CI 0.03–0.77], 3 months RR 0.14 [95% CI 0.03–0.77], and 5 years RR 0.43 [95% CI 0.20–0.94]). Supplementary figure S5 shows these in more detail (Additional file 5). There was no significant difference in renal function improvement, as shown in Supplementary figure S6 (Additional file 5). The serum creatinine level was significantly lower in the PE group at 5 years as shown in Supplementary figure S7 (Additional file 5). Relapse RR was 0.81 (95% CI 0.55–1.18) in the overall observational period (Fig. 3 and Supplementary figure S8 in Additional file 5). Only one paper evaluated the QOL index as + 1.08 (95% CI − 0.46–2.62) for the physical component of SF-36 and + 0.54 (95% CI − 0.67–1.75) for the mental component (Fig. 3). The results measured with EQ-5D, as shown in Supplementary figure S9, were similar (Additional file 5).

### PE vs. pulse steroid treatment

#### Included studies

One RCT on PE vs. pulse steroid treatment involving 137 AAV patients (median age 66 years, 38.7% of females) was included (Table 2). Short- and long-term prognoses were reported in separate papers [26, 27]. Included patients had MPA or GPA. The PE method was albumin replacement, and the pulse steroid treatment was intravenous methylprednisolone 1000 mg/day for 3 days. The maximum observational period was 10 years (median 3.95 years) as shown in Supplementary Table S1 (Additional file 5). The positive rates for C-ANCA and P-ANCA were 43% and 52%, respectively. The prevalence of renal lesions was 100%, but there was little information on other organs. As a combination therapy, prednisolone, cyclophosphamide, and azathioprine were used for induction or maintenance of remission in both groups.

**Table 2:** Summary of included PE vs pulse steroid treatment RCT in this review

Source	Inclusion criteria	Exclusion criteria	No. of patients	Interventions	Primary outcome
MEPEX 2007, 2013 [26, 27]	A diagnosis of WG or MPA; biopsy proven, pauci-immune, necrotizing, and/or crescentic glomerulonephritis, in the absence of other glomerulopathy; serum creatinine > 500 μmol/L.	Age < 18 or > 80 years; inadequate contraception in women of childbearing age; pregnancy; previous malignancy; HBV antigenaemia, anti-HCV, or anti-HIV antibody; other multisystem autoimmune disease; anti-GBM disease; life-threatening non-renal manifestations of vasculitis, including alveolar haemorrhage requiring mechanical ventilation within 24 h of admission; dialysis for > 2 weeks before entry; creatinine> 200 mol/L > 1 year before entry; a second clearly defined cause of renal failure; previous episode of biopsy-proven necrotizing and/or crescentic glomerulonephritis; > 2 weeks of treatment with cyclophosphamide or azathioprine; > 500 mg of intravenous methylprednisolone; PE within the preceding year; > 3 months of treatment with oral prednisolone; allergy to study medications.	137, MPA (69%) or GPA (31%)	PE^†^ [(a) centrifugation or filter separation, (b) 5% albumin, (c) 60 mL/kg, (d) 7 sessions within 14 days] vs pulse steroid treatment, [intravenous methylpredonisolone 1000 mg/day for 3 days]	Renal recovery at 3 months

*RCT* randomised controlled trial, *PE* plasma exchange, *WG* Wegener’s granulomatosis, *MPA* microscopic polyangiitis, *HBV* hepatitis B virus, *HCV* hepatitis C virus, *HIV* human immunodeficiency virus, *GBM* glomerular basement membrane, *GPA* granulomatosis with polyangiitis

^†^The detailed methods of treatments are described in parentheses. In PE, (a) separation method, (b) replacement fluid, (c) dose per session, and (d) number of sessions

#### Clinical outcomes

**Fig. 4:**
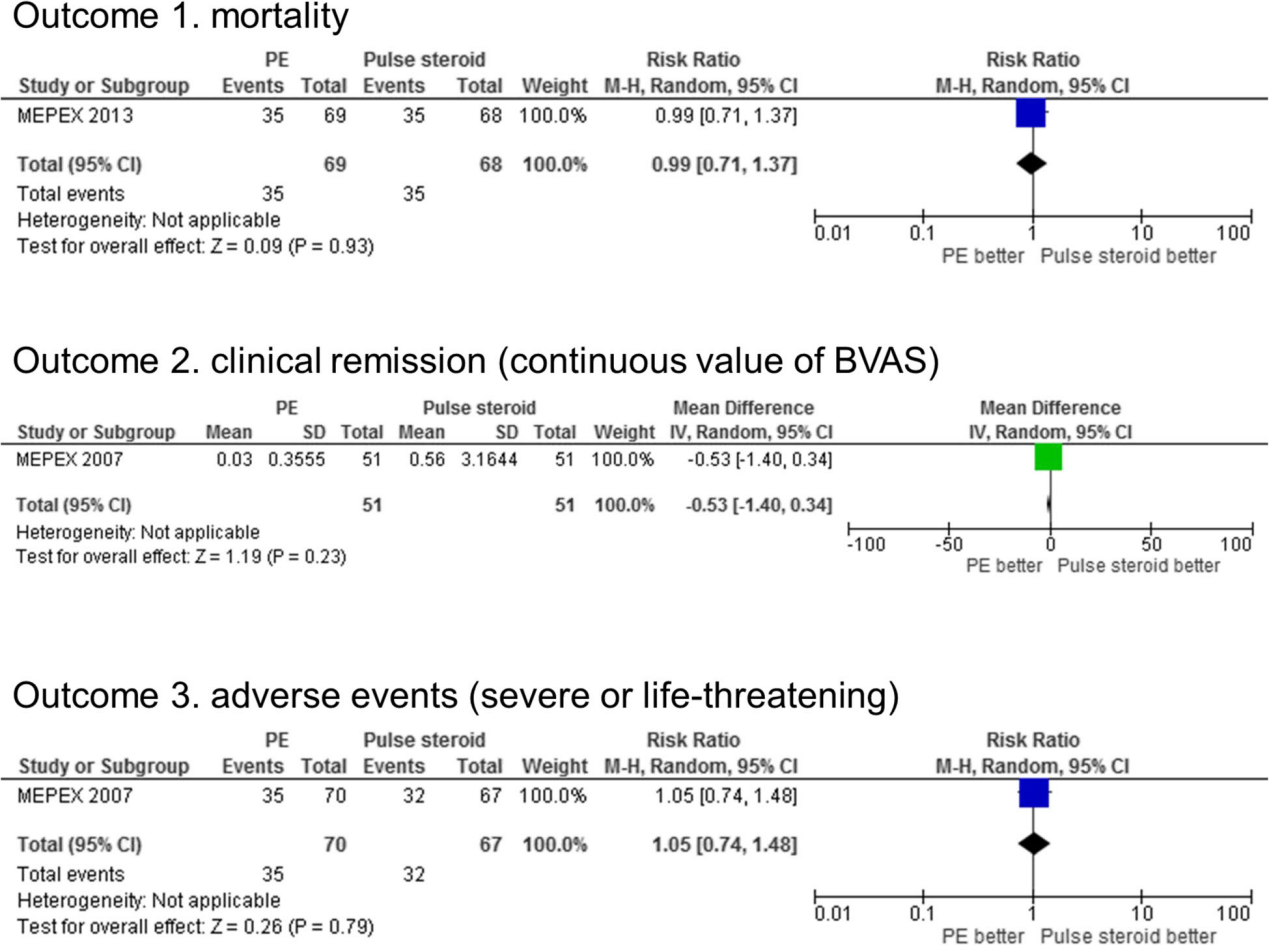
Forrest plots of primary outcomes between the PE and pulse steroid treatment groups. Regarding the timing of outcome measurement in this figure, mortality and adverse events are for the overall observational period, and clinical remission (BVAS) is at 1 year after the allocation. See Additional file 5 for other data. In this figure, as adverse events, data of only severe or life-threatening cases are displayed. Abbreviations: PE, plasma exchange; BVAS, Birmingham Vasculitis Activity Score

Throughout the overall observational period, a total of 35 (51%) and 35 (51%) deaths occurred in the PE and pulse steroid groups, respectively (Fig. 4), with no statistically significant difference. The mortality RR was 0.96 (95% CI, 0.45–2.06) and 1.14 (95% CI 0.64–2.02) after 3 months and 1 year, respectively, as shown in Supplementary figure S10 (Additional file 5).

Regarding CR (Fig. 4), BVAS was considered a continuous value. BVAS after 1 year was 0.03 ± 0.36 and 0.56 ± 3.16 in the PE and pulse steroid groups, respectively, with no significant difference. After 3 months, the mean difference was − 1.09 (95% CI − 2.34–0.16) as shown in Supplementary figure S11 (Additional file 5).

A total of 35 (50%) and 32 (48%) severe or life-threatening AEs occurred in the PE and pulse steroid groups, respectively (Fig. 4). A total of 63 (90%) and 59 (88%) mild-to-moderate AEs were reported in the PE and pulse steroid groups, respectively, with no statistically significant difference as shown in Supplementary figure S12 (Additional file 5).

The composite ESRD or death RR was 0.86 (95% CI 0.66–1.11) during the overall observational period in the PE vs. pulse steroid groups comparison as shown in Supplementary figure S13 (Additional file 5). ESRD (death-censored) RR was 0.69 (95% CI 0.45–1.04) during the overall observational period as shown in Supplementary figure S14 (Additional file 5). The risk of developing ESRD was significantly lower in the PE group at 3 months (RR 0.46 [95% CI 0.24–0.86]) and 1 year (RR 0.44 [95% CI 0.22–0.85]) after allocation. There was a statistically significant improvement in renal function at 3 months in the PE group (RR 1.35 [95% CI 1.04–1.86]), as shown in Supplementary figure S15 (Additional file 5). The serum creatinine level, relapse risk, and VDI were not significantly different between the two groups as shown in additional figure files (Supplementary figure S16–18 in Additional file 5). In MEPEX [26], evaluation of SF-36 scores revealed no significant differences between groups. However, there was insufficient data to calculate the mean difference.

### Risk of bias in individual studies

The risk of bias item was assessed across included studies as shown in Supplementary figure S19 and S20 (Additional file 5). As shown on the graph, regarding studies on PE vs. no PE, studies by Zäuner et al. [31] on mortality, Szpirt et al. [28] on CR, Pusey et al. [30] and Zäuner et al. [31] on AEs, and PEXIVAS [17] on QOL were judged as having a high risk of bias in the ‘overall’ domain. Regarding PE vs. pulse steroid, studies by MEPEX [26, 27] on CR, QOL, and VDI were judged as having a high risk of bias. The other studies had some concerns.

### Subgroup analysis and sensitivity analysis

First, we divided the studies on PE vs. no PE by type of AAV (only MPA or only GPA) as shown in Supplementary figure S21 (Additional file 5). Mortality RR was similar between the two groups (*P* value for interaction = 0.33). Next, regarding PE condition, we divided studies on PE vs. no PE into albumin preparation or fresh frozen plasma replacement groups as shown in Supplementary figure S22 (Additional file 5). Mortality RR was similar between the two groups (*P* value for interaction = 0.55). We could not perform a subgroup analyses of either CR or AE due to the lack of data or of ANCA status and localisation of lesions, since there were no studies available.

The results of the sensitivity analysis were similar to the results of the primary analysis as shown in Supplementary figure S23 and table S2, (Additional file 5).

## Discussion

We conducted a review of multiple RCTs investigating the efficacy of PE for AAV. This is a novel review in that the search strategy covers all three AAV types, the target population was not limited to those with renal lesions, and the results of the latest and largest RCT, PEXIVAS, were included.

In the current review, when comparing the incidence of primary outcomes between PE and non-PE groups (both PE vs. no PE and PE vs. pulse steroid), there was no significant difference. The latest observational study using real-world data also showed similar results that the additional PE had no benefit [35]. Although there are lots of pathological dogmas that AAV are autoimmune diseases driven by ANCA, this result could raise a question of whether ANCA plays a major role in the pathogenesis of AAV, since PE directly removes ANCA from patients’ serum. There is also evidence suggesting that ANCA might not be majorly involved in the pathogenesis of AAV. First, most patients with ANCA do not develop AAV [36]. Second, the correlation between ANCA titers and the disease activity of AAV remains elusive [37]. Third, ANCA might not be the main reason for the forward loop of NETosis fuelling inflammation in AAV and could just be a marker of leukocytoclastic inflammation in small blood vessels [38]. Forth, improvement of AAV by rituximab could better fit with the hypothesis that circulating B cell plays an important pathogenic role, rather than plasmocytes, which is the major source of antibodies including ANCA [39]. Fifth, cyclophosphamide and its metabolites also target endothelial cells, which might not only be the victims in AAV but also the culprits [40]. Further elucidation of the pathophysiology of AAV is desired in the future. On the other hand, as a remarkable point with the secondary outcomes, PE was associated with a statistically significant reduction in the ESRD (death-censored) development rate in the early stage of treatment. These results were observed in both PE vs. no PE and PE vs. pulse steroid analyses. There is a possibility that a selection bias may be involved in this reduction in ESRD rate because recent data (< 1 year after allocation in PEXIVAS, which is the largest RCT in this review) were not available. However, the Kaplan-Meier curve showing the incidence of the primary endpoint (ESRD or death) in the PEXIVAS report [17] indicated fewer occurrences of the endpoints in the PE group than in the no PE group in the early stage of treatment. In the Kaplan-Meier plot, the two curves met at 2–3 years after the start of observation. Therefore, there is an impression that the results of PEXIVAS were consistent with those of other studies. PE might suppress early renal injury by removing humoral factors that exacerbate renal injury of AAV.

Although PE has long been used as one of the treatment options for AAV, so far, its therapeutic indication needs to be thoroughly considered once again based on the results of this review. It is not recommended to actively perform PE for all AAV patients, because PE showed no benefit in terms of the primary outcomes of this study. Further, there are other disadvantages, including high treatment costs [41]. On the other hand, PE may be a treatment option for AAV patients with a high risk of early renal failure, since it suppressed ESRD in the early stage of treatment. However, patients who developed ESRD in the early stage of treatment (at 3 months; PE vs. no PE) were only 24% (10/41) of the no PE group and merely 13% (11/84) of all patients. It could be worth to retrospectively study the baseline profile of patients who developed early renal failure and responded to treatment to try and restrict the use of PE in such patients. Moreover, since the statistically significant difference of ESRD between the two groups disappeared in the long run, it needs to be evaluated whether early stage-limited ESRD suppression is clinically meaningful from various viewpoints, such as QOL and treatment cost.

The search results of this review revealed that there was no RCT that evaluated the effect of PE on EGPA. Although Guillevin et al. performed RCTs, including CSS (currently EGPA) patients, only approximately 20% of the participants had CSS, while a majority of the patients had polyarteritis nodosa [42, 43]. Therefore, evaluating the efficacy of PE for CSS from those results was considered difficult; thus, those studies were excluded from the current review. As a side note, Guillevin et al. extracted the data of 32 patients diagnosed with MPA and CSS (28 MPA, 4 CSS) from the results of these RCTs and performed a sub-analysis that integrated the results [34]. They also concluded that PE has no added benefit. An RCT in a large population of EGPA patients is required in the future.

This is the first review after the detailed results of PEXIVAS were published [17]. Although PEXIVAS targeted patients with severe AAV, only 14% (99/704) of the patients died and only 20% (138/704) reached ESRD during the observation period. Further, fewer hard outcomes were noted than those in other RCTs. In this review, we were able to analyse not only the primary outcome but also other secondary outcomes of PEXIVAS such as relapse and QOL indicators, and the breakdown of AEs in more detail compared to other previous reviews.

Since the diagnostic criteria of vasculitis syndrome have changed over time [6, 7], when conducting a systematic review, sufficient attention is necessary for setting the eligibility criteria. In this review, we set the eligibility criteria to include only patients with a definite diagnosis of AAV (MPA, GPA, or EGPA [+RLV]), similar to another previous review protocol [44]. Therefore, patients having old diagnoses, such as idiopathic rapidly progressive glomerulonephritis and idiopathic crescentic glomerulonephritis [33, 45], which do not meet the current diagnostic criteria and that may include non-AAV patients, were excluded [46–50]. Therefore, we could reduce the possibility that non-AAV patients were included in this review, and that the results are now more reliable and easier for the clinicians to understand.

This review has limitations. Local search sources, such as Japanese and Chinese sources, were not searched. In addition, since 2 of the included studies included < 20% ineligible patients, the PE vs. no PE meta-analysis included only a few patients (10 [1.2%]) who were not diagnosed with AAV. These might have involved a selection bias. Finally, the definitions of CR and improvement in renal function differed among studies, which might involve an information bias.

## Conclusions

We performed the latest review to assess the efficacy of PE for AAV, which included all AAV subtypes (MPA, GPA, and EGPA) in the search scope. In AAV patients, performing PE was not significantly associated with the risk of primary outcomes, mortality, CR, and AEs. In the secondary outcomes, it was suggested that PE may be effective in suppressing ESRD in the early stages of treatment. None of the RCTs verified the effect of PE on EGPA, and this should be investigated in the future.

## Supplementary Information

**Additional file 1.** PRISMA 2009 checklist.

**Additional file 2.** The pre-specified study protocol of this review.

**Additional file 3.** The search strategy used in this review.

**Additional file 4.** The result of the 39 full-text records assessed for eligibility.

**Additional file 5.** Supplementary tables and figures.

## Data Availability

The datasets used and/or analysed during the current study are available from the corresponding author on reasonable request.

## Abbreviations

AAV: ANCA-associated vasculitis
AEs: Adverse events
ANCA: Antineutrophil cytoplasmic antibody
BVAS: Birmingham Vasculitis Activity Score
CI: Confidence interval
CR: Clinical remission
CSS: Churg-Strauss syndrome
CYC: Cyclophosphamide
EGPA: Eosinophilic granulomatosis with polyangiitis
ESRD: End-stage renal disease
GBM: Glomerular basement membrane
GC: Glucocorticoid
GPA: Granulomatosis with polyangiitis
HBV: Hepatitis B virus
HCV: Hepatitis C virus
HIV: Human immunodeficiency virus
IRPGN: Idiopathic rapidly progressive glomerulonephritis
MPA: Microscopic polyangiitis
NETs: Neutrophil extracellular trap
PE: Plasma exchange
PRISMA: Preferred Reporting Items for Systematic Reviews and Meta-Analysis
PROSPERO: International Prospective Register of Systematic Reviews
QOL: Health-related quality of life
RCTs: Randomised controlled trials
RLA: Renal-limited vasculitis
RPGN: Rapidly progressive glomerulonephritis
RR: Risk ratio
UMIN-CTR: University Hospital Medical Information Network Clinical Trials Registry
VDI: Vasculitis Damage Index
WG: Wegener’s granulomatosis
