# Supplementary material for: Efficacy of plasma exchange for antineutrophil cytoplasmic antibody-associated systemic vasculitis: a systematic review and meta-analysis
Source: Arthritis Res Ther. 2021 Jan 14;23:28. doi: 10.1186/s13075-021-02415-z (PMC7809754; doi:10.1186/s13075-021-02415-z)
Supplement: Supplementary file 2 — Additional file 2. The pre-specified study protocol of this review. [file 13075_2021_2415_MOESM2_ESM.docx]

**Additional file 2. The pre-specified study protocol of this review.**

**Title: Plasma exchange for ANCA-associated vasculitis: protocol for a systematic review and meta-analysis**

**Introduction**

Anti-neutrophil cytoplasmic antibody (ANCA)-associated vasculitis (AAV) is a systemic condition characterized by ANCA production and serum positivity that injures small- to medium-sized blood vessels in organs throughout the body. (1) AAV often presents as rapidly progressive glomerulonephritis in the kidneys and interstitial pneumonia or alveolar haemorrhage, all of which are life-threatening. AAV includes the following types of diseases: microscopic polyangiitis (MPA), granulomatosis with polyangiitis (GPA), eosinophilic granulomatosis with polyangiitis (EGPA), and renal-limited vasculitis (RLV). RLV is interpreted as a subtype of MPA in some papers and guidelines. (2-5) AAV is caused by autoimmune mechanisms. In afflicted patients, the main ANCA target antigens are myeloperoxidase and proteinase 3. (6) Treatment for AAV is typically with immunosuppressive agents, such as cyclophosphamide, rituximab and combined glucocorticoid therapy, for induction immunosuppressive therapy. (7) Recently, ANCA has been reported to activate neutrophils directly, which then adhere to and penetrate vessel walls. (8) The activated neutrophils release various inflammatory mediators and factors that stimulate the alternative complement pathway, after which generated complement 5a amplifies inflammation by recruiting more neutrophils. (9) The activated neutrophils also develop a neutrophil extracellular trap. (10) As ANCA itself appears to associate with the progression of severe vasculitis lesions, eliminating ANCA and its various mediators by plasma exchange (PE) may represent an effective addition to immunosuppressive therapy for patients with AAV. (11) However, PE requires large amounts of fresh frozen plasma and albumin preparation, which creates a risk of hypocalcaemia, hypo- or hypervolemia and anaphylactoid reactions. (12) Several randomized control studies have investigated the efficacy and safety of PE for AAV. The results of the largest trial to date, the PEXIVAS study (13), were also published in February 2020. However, findings have been inconsistent among the studies. It remains uncertain whether PE can improve the prognosis of afflicted patients.

**Objectives**

Assess if PE improves prognosis in AAV patients.

**Methods and analysis**

*Study design and protocol*

This protocol follows the Preferred Reporting Items for Systematic Review and Meta-Analysis Protocols (PRISMA-P) statements (14). (see PRISMA-P checklist attached on the last of this file.)

*Eligibility criteria for considering studies for this review*

Studies that meet the following criteria will be included in this review:

- Types of studies

We will consider only randomized controlled trials (RCTs) for inclusion in this review.

- Participants

Inclusion criteria: All studies primarily concerning AAV in adult populations (i.e., 18 years of age or older). Diagnosis of AAV including confirmed GPA (formerly Wegener's granulomatosis; WG), EGPA (formerly Churg-Strauss syndrome; CSS), MPA and RLV. (15)

Exclusion criteria: Patients with other types of vasculitis.

- Intervention and comparator

This review will consider studies that evaluate the effectiveness of PE in AAV.

Intervention: PE group

Comparator: Non-PE or sham PE group

We will include any method or dose of PE treatment. In reference to the Guidelines of the American Society for Apheresis, PE is defined as “a therapeutic procedure in which blood of the patient is passed through a medical device which separates plasma from other components of blood. The plasma is removed and replaced with a replacement solution such as colloid solution (e.g., albumin and/or plasma) or a combination of crystalloid/colloid solution.” (16) Therefore, typical PE, double filtration plasmapheresis (17), and selective PE (18) will be included. A subgroup analysis considering the difference among these separation techniques will also be performed. Immunoabsorption treatment will be excluded.

The purpose of this review is to evaluate the effect of eliminating ANCA and its various mediators by PE from the pathophysiological point of view. This work also aims to evaluate the effect of PE as an add-on to conventional treatment. Therefore, we will include studies whose design can evaluate the effect of PE alone, i.e., PE + treatment A compared with non-PE (or sham PE) + treatment A. If PE + treatment A is compared with non-PE (or sham PE) + no other treatment or if PE is compared directly with another treatment, we will assess those results separately as additional analyses.

- Types of outcome measures

Primary outcomes:

1. Mortality

2. Clinical remission (as defined by the study’s authors, typically as the complete absence of disease activity determined by the Birmingham Vasculitis Activity Score) (19)

3. Adverse events (PE- and non-PE-related events)

Secondary outcomes:

1. Renal failure (end-stage renal disease [induction of haemodialysis, peritoneal dialysis, or kidney transplantation] or changes in serum creatinine level or estimated glomerular filtration rate)

2. Disease flare/relapse (as defined by the study’s authors, typically as increased disease activity from a previously low or absent state)

3. Health-related quality of life (as assessed by the Short Form-36 or other health-related quality of life measures, including those specific to AAV) (20)

4. Disease damage according to the Vasculitis Damage Index, the AAV Index of Damage or other validated disease damage scores accepted by Outcome Measures in Rheumatology (21, 22)

Time points: We will collect data at six months, at 12 months and after 12 months as the major time points for measuring outcomes.

In the case of missing data, we will contact the authors and clarify the primary studies if required; such inclusions will be reported in the review.

*Report characteristics*

We will apply no restrictions on language, length of follow-up, publication status or date of publication.

*Information sources*

We will search the following electronic databases and sources to identify studies:

- PubMED
- MEDLINE
- Embase
- Cochrane Central Register of Controlled Trials

We will search the following ongoing trial registries:.

- ClinicalTrials.gov (https://www.clinicaltrials.gov/)
- World Health Organization trials portal (https://www.who.int/ictrp/en/)

When we identify unpublished trials, we will contact the authors for further information.

We will check the reference lists of all primary studies and review articles for additional references.

*Search strategy*

See “Additional file 3”

*Data management*

The searched results will be managed using EndNote X9 (Clarivate Analytics Co., Philadelphia, United States).

*Selection process*

For study selection, two review authors (YY, MH or YH) will independently screen titles and abstracts of articles identified by the search to determine their potential for inclusion in the review. Those articles will be coded as “yes/maybe” (eligible/potentially eligible or unclear) or “no” (ineligible). We will retrieve full-text study reports/publications of the articles coded as “yes/maybe”, and the two review authors will independently screen them to identify studies for inclusion. The reasons for excluding ineligible studies will be recorded. Disagreements will be resolved through discussion or consultation with a third review author (KH or YK) if required. We will identify and exclude duplicates and collate multiple reports of the same study so that each study, rather than each report, will be a unit of interest in the review.

The results of the search and the full process for selecting included studies will be presented in a PRISMA flow diagram. (23)

*Data collection process*

Data extraction from studies included in the review will be performed by two independent review authors (YY, MH or YH) using a prepared data extraction form. For each study, the author names, place and year of publication, data on sample size and characteristics, characteristics of interventions performed, instruments used to assess outcomes and results will be collected. The data extraction form will be piloted before commencing data collection. Any disagreements that arise between the reviewers will be resolved through discussion or with a third reviewer (KH or YK). The collected data will be transferred to Review Manager 5, Version 5.3 (Copenhagen, Nordic Cochrane Centre, Germany).

*Risk of bias in individual studies*

We will assess the risk of bias in the included studies using Cochrane's domain-based evaluation tool as recommended in the *Cochrane Handbook for Systematic Reviews of Interventions* (https://training.cochrane.org/handbook/PDF/v6). We will use the Cochrane 'Risk of bias' tool 2 (24) to evaluate the following domains of bias: bias arising from the randomization process, bias due to deviation from intended intervention, bias due to missing outcome data, bias in measurement of the outcome and bias in selection of the reported result. Two review authors (YY, MH or YH) will independently assess the risk of bias for each domain as low risk of bias, some concerns, or high risk of bias. Discrepancies will be resolved by discussion, and a third review author (KH or YK) will be consulted whenever necessary to make a final judgment.

*Data synthesis*

The data will be synthesized qualitatively, focusing on the comparability and characteristics of studies that may affect the cumulative evidence. A meta-analysis will be undertaken when deemed appropriate with a random-effects model due to the expected variation observed among studies. Dichotomous data will be analyzed using risk ratios with 95% confidence intervals (CIs). Continuous data will be analyzed as mean differences with 95% CIs when measurements are made using the same scale, or as standardized mean differences with 95% CIs when measurements are obtained using different scales.

For assessment of heterogeneity, clinical heterogeneity will first be evaluated by comparing the characteristics of individual studies (i.e., study participants, eligibility criteria and outcomes). Methodologic heterogeneity will be assessed by comparing design characteristics, including risk of bias assessments of individual studies. We will utilize the I^2^ statistic to determine statistical heterogeneity across included studies. When significant, clinical, methodologic or statistical heterogeneity (defined as I^2^ statistic above 50%) (25) is detected, possible causes will be explored by prespecified subgroup and sensitivity analysis.

*Subgroup analysis*

When sufficient studies and data exist, we will undertake the following subgroup analyses:

- Type of AAV (MPA, EGPA [CSS], GPA [WG] or RLV)
- ANCA status (P-ANCA, C-ANCA, ANCA-negative and difference in ANCA titer)
- PE conditions (volume of plasma exchanged, number and frequency of sessions, nature of the replacement solution and separation technique)
- Localization of lesions (presence of renal, lung or nerve lesion or other)

*Sensitivity analysis*

We plan to carry out the following sensitivity analyses on primary outcomes to determine if a high risk of bias in some included studies affected the study results:

- Exclusion of studies at high risk of bias
- Exclusion of trials with 10 or fewer events
- Exclusion of cluster RCTs
- Comparing fixed-effect pooled estimates or 95% CIs versus random-effect pooled estimates or 95% CIs

*Assessment of reporting bias*

We will attempt to reduce reporting bias by searching the grey literature for unpublished studies. If 10 or more studies are included, we will check for the existence of publication bias using funnel plots.

**Declarations**

*Conflict of interests*

The authors declare that they have no conflict of interests.

*Funding*

The authors have not received any funding in relation to this review.

*Availability of data and materials*

Not applicable

*Ethics approval and consent to participate*

The data presented in this review will not contain individual patient data. Therefore, ethical approval is not required.

*Consent for publication*

Not applicable

*Dissemination plan*

We will disseminate the results of this systematic review by publication in a peer-reviewed journal and presentation at relevant medical conferences.

*Stage of this review*

This study was registered at the University Hospital Medical Information Network Clinical Trials Registry (UMIN-CTR) as UMIN R000045239 on March 6, 2020 (<https://upload.umin.ac.jp/cgi-open-bin/ctr_e/ctr_view.cgi?recptno=R000045239>). As of the submission of this proposal, we have performed preliminary searches and piloting of the study selection process but have not begun the formal screening of search results against eligibility criteria.

*Author contributions*

YY and MH contributed to this paper equally. YY, MH, YH and KH developed the research question. YY, MH and SY wrote the first draft of the manuscript. YY, MH, YH, SY and KH contributed to development of methods, search strategies and writing of this manuscript. KY contributed to the refinement of the manuscript. All authors contributed to drafting the review protocol and have approved the final manuscript.

**References**

1. Pagnoux C. Updates in ANCA-associated vasculitis. Eur J Rheumatol. 2016;3(3):122-33.

2. Watts R, Lane S, Hanslik T, Hauser T, Hellmich B, Koldingsnes W, et al. Development and validation of a consensus methodology for the classification of the ANCA-associated vasculitides and polyarteritis nodosa for epidemiological studies. Ann Rheum Dis. 2007;66(2):222-7.

3. Ntatsaki E, Carruthers D, Chakravarty K, D'Cruz D, Harper L, Jayne D, et al. BSR and BHPR guideline for the management of adults with ANCA-associated vasculitis. Rheumatology (Oxford). 2014;53(12):2306-9.

4. Harigai M, Nagasaka K, Amano K, Bando M, Dobashi H, Kawakami T, et al. 2017 Clinical practice guidelines of the Japan Research Committee of the Ministry of Health, Labour, and Welfare for Intractable Vasculitis for the management of ANCA-associated vasculitis. Mod Rheumatol. 2019;29(1):20-30.

5. Yates M, Watts RA, Bajema IM, Cid MC, Crestani B, Hauser T, et al. EULAR/ERA-EDTA recommendations for the management of ANCA-associated vasculitis. Ann Rheum Dis. 2016;75(9):1583-94.

6. Jennette JC, Nachman PH. ANCA Glomerulonephritis and Vasculitis. Clinical journal of the American Society of Nephrology : CJASN. 2017;12(10):1680-91.

7. Specks U, Merkel PA, Seo P, Spiera R, Langford CA, Hoffman GS, et al. Efficacy of remission-induction regimens for ANCA-associated vasculitis. The New England journal of medicine. 2013;369(5):417-27.

8. Jennette JC, Falk RJ. Pathogenesis of antineutrophil cytoplasmic autoantibody-mediated disease. Nat Rev Rheumatol. 2014;10(8):463-73.

9. Xiao H, Schreiber A, Heeringa P, Falk RJ, Jennette JC. Alternative complement pathway in the pathogenesis of disease mediated by anti-neutrophil cytoplasmic autoantibodies. Am J Pathol. 2007;170(1):52-64.

10. Soderberg D, Segelmark M. Neutrophil Extracellular Traps in ANCA-Associated Vasculitis. Front Immunol. 2016;7:256.

11. Winters JL. Plasma exchange: concepts, mechanisms, and an overview of the American Society for Apheresis guidelines. Hematology Am Soc Hematol Educ Program. 2012;2012:7-12.

12. Mokrzycki MH, Kaplan AA. Therapeutic plasma exchange: complications and management. American journal of kidney diseases : the official journal of the National Kidney Foundation. 1994;23(6):817-27.

13. Walsh M, Merkel PA, Peh C-A, Szpirt WM, Puéchal X, Fujimoto S, et al. Plasma Exchange and Glucocorticoids in Severe ANCA-Associated Vasculitis. New England Journal of Medicine. 2020;382(7):622-31.

14. Moher D, Shamseer L, Clarke M, Ghersi D, Liberati A, Petticrew M, et al. Preferred reporting items for systematic review and meta-analysis protocols (PRISMA-P) 2015 statement. Systematic Reviews. 2015;4(1):1.

15. Jennette JC, Falk RJ, Bacon PA, Basu N, Cid MC, Ferrario F, et al. 2012 revised International Chapel Hill Consensus Conference Nomenclature of Vasculitides. Arthritis Rheum. 2013;65(1):1-11.

16. Schwartz J, Padmanabhan A, Aqui N, Balogun RA, Connelly-Smith L, Delaney M, et al. Guidelines on the Use of Therapeutic Apheresis in Clinical Practice–Evidence-Based Approach from the Writing Committee of the American Society for Apheresis: The Seventh Special Issue. Journal of Clinical Apheresis. 2016;31(3):149-338.

17. Mineshima M. Double filtration plasmapheresis: Determination of the optimal albumin concentration in the supplementation fluid. Transfusion and apheresis science : official journal of the World Apheresis Association : official journal of the European Society for Haemapheresis. 2017;56(5):654-6.

18. Ohkubo A, Okado T. Selective plasma exchange. Transfusion and apheresis science : official journal of the World Apheresis Association : official journal of the European Society for Haemapheresis. 2017;56(5):657-60.

19. Luqmani RA, Bacon PA, Moots RJ, Janssen BA, Pall A, Emery P, et al. Birmingham Vasculitis Activity Score (BVAS) in systemic necrotizing vasculitis. Qjm. 1994;87(11):671-8.

20. Busija L, Pausenberger E, Haines TP, Haymes S, Buchbinder R, Osborne RH. Adult measures of general health and health-related quality of life: Medical Outcomes Study Short Form 36-Item (SF-36) and Short Form 12-Item (SF-12) Health Surveys, Nottingham Health Profile (NHP), Sickness Impact Profile (SIP), Medical Outcomes Study Short Form 6D (SF-6D), Health Utilities Index Mark 3 (HUI3), Quality of Well-Being Scale (QWB), and Assessment of Quality of Life (AQoL). Arthritis care & research. 2011;63 Suppl 11:S383-412.

21. Exley AR, Bacon PA, Luqmani RA, Kitas GD, Gordon C, Savage CO, et al. Development and initial validation of the Vasculitis Damage Index for the standardized clinical assessment of damage in the systemic vasculitides. Arthritis Rheum. 1997;40(2):371-80.

22. Merkel PA, Aydin SZ, Boers M, Direskeneli H, Herlyn K, Seo P, et al. The OMERACT core set of outcome measures for use in clinical trials of ANCA-associated vasculitis. J Rheumatol. 2011;38(7):1480-6.

23. Moher D, Liberati A, Tetzlaff J, Altman DG. Preferred reporting items for systematic reviews and meta-analyses: the PRISMA statement. PLoS Med. 2009;6(7):e1000097.

24. Sterne JAC, Savovic J, Page MJ, Elbers RG, Blencowe NS, Boutron I, et al. RoB 2: a revised tool for assessing risk of bias in randomised trials. BMJ (Clinical research ed). 2019;366:l4898.

25. Higgins JPT, Thompson SG. Quantifying heterogeneity in a meta-analysis. Statistics in Medicine. 2002;21(11):1539-58.

**PRISMA-P 2015 Checklist**

This checklist has been adapted for use with protocol submissions to *Systematic Reviews* from Table 3 in Moher D et al**:** Preferred reporting items for systematic review and meta-analysis protocols (PRISMA-P) 2015 statement. *Systematic Reviews* 2015 **4**:1

| **Section/topic** | **#** | **Checklist item** | **Information reported** | | **Line number(s)** |
| --- | --- | --- | --- | --- | --- |
|  |  |  | **Yes** | **No** |  |
| **ADMINISTRATIVE INFORMATION** | | | | | |
| **Title** | | | | | |
| Identification | 1a | Identify the report as a protocol of a systematic review |  |  | 3,4 |
| Update | 1b | If the protocol is for an update of a previous systematic review, identify as such |  |  | NA |
| **Registration** | 2 | If registered, provide the name of the registry (e.g., PROSPERO) and registration number in the Abstract |  |  | 201-206 |
| **Authors** | | | | | |
| Contact | 3a | Provide name, institutional affiliation, and e-mail address of all protocol authors; provide physical mailing address of corresponding author |  |  | In main text. |
| Contributions | 3b | Describe contributions of protocol authors and identify the guarantor of the review |  |  | 208-213 |
| **Amendments** | 4 | If the protocol represents an amendment of a previously completed or published protocol, identify as such and list changes; otherwise, state plan for documenting important protocol amendments |  |  | NA |
| **Support** | | | | | |
| Sources | 5a | Indicate sources of financial or other support for the review |  |  | 184-185 |
| Sponsor | 5b | Provide name for the review funder and/or sponsor |  |  | NA |
| Role of sponsor/funder | 5c | Describe roles of funder(s), sponsor(s), and/or institution(s), if any, in developing the protocol |  |  | NA |
| **INTRODUCTION** | | | | | |
| **Rationale** | 6 | Describe the rationale for the review in the context of what is already known |  |  | 6-29 |
| **Objectives** | 7 | Provide an explicit statement of the question(s) the review will address with reference to participants, interventions, comparators, and outcomes (PICO) |  |  | 31-32 |
| **METHODS** | | | | | |
| **Eligibility criteria** | 8 | Specify the study characteristics (e.g., PICO, study design, setting, time frame) and report characteristics (e.g., years considered, language, publication status) to be used as criteria for eligibility for the review |  |  | 39-65 |
| **Information sources** | 9 | Describe all intended information sources (e.g., electronic databases, contact with study authors, trial registers, or other grey literature sources) with planned dates of coverage |  |  | 90-100 |
| **Search strategy** | 10 | Present draft of search strategy to be used for at least one electronic database, including planned limits, such that it could be repeated |  |  | 102-103 |
| ***STUDY RECORDS*** | | | | | |
| Data management | 11a | Describe the mechanism(s) that will be used to manage records and data throughout the review |  |  | 105-107 |
| Selection process | 11b | State the process that will be used for selecting studies (e.g., two independent reviewers) through each phase of the review (i.e., screening, eligibility, and inclusion in meta-analysis) |  |  | 109-120 |
| Data collection process | 11c | Describe planned method of extracting data from reports (e.g., piloting forms, done independently, in duplicate), any processes for obtaining and confirming data from investigators |  |  | 122-130 |
| **Data items** | 12 | List and define all variables for which data will be sought (e.g., PICO items, funding sources), any pre-planned data assumptions and simplifications |  |  | 122-127 |
| **Outcomes and prioritization** | 13 | List and define all outcomes for which data will be sought, including prioritization of main and additional outcomes, with rationale |  |  | 66-82 |
| **Risk of bias in individual studies** | 14 | Describe anticipated methods for assessing risk of bias of individual studies, including whether this will be done at the outcome or study level, or both; state how this information will be used in data synthesis |  |  | 132-141 |
| ***DATA*** | | | | | |
| **Synthesis** | 15a | Describe criteria under which study data will be quantitatively synthesized |  |  | 144,145 |
|  | 15b | If data are appropriate for quantitative synthesis, describe planned summary measures, methods of handling data, and methods of combining data from studies, including any planned exploration of consistency (e.g., *I* ^2^, Kendall’s tau) |  |  | 143-157 |
|  | 15c | Describe any proposed additional analyses (e.g., sensitivity or subgroup analyses, meta-regression) |  |  | 159-174 |
|  | 15d | If quantitative synthesis is not appropriate, describe the type of summary planned |  |  | 144,145 |
| **Meta-bias(es)** | 16 | Specify any planned assessment of meta-bias(es) (e.g., publication bias across studies, selective reporting within studies) |  |  | 176-178 |
| **Confidence in cumulative evidence** | 17 | Describe how the strength of the body of evidence will be assessed (e.g., GRADE) |  |  | NA |
