# Supplementary material for: Efficacy of plasma exchange for antineutrophil cytoplasmic antibody-associated systemic vasculitis: a systematic review and meta-analysis
Source: Arthritis Res Ther. 2021 Jan 14;23:28. doi: 10.1186/s13075-021-02415-z (PMC7809754; doi:10.1186/s13075-021-02415-z)
Supplement: Supplementary file 3 — Additional file 3. The search strategy used in this review. [file 13075_2021_2415_MOESM3_ESM.docx]

**Additional file 3. The search strategy used in this review.**

Results 17-6-2020:

- PubMed: 244
- MEDLINE: 245
- Embase: 442
- COCHRANE Library (CENTRAL): 162

Databases:

**PubMed**

<http://www.ncbi.nlm.nih.gov/pubmed?otool=leiden>

<https://pmlegacy.ncbi.nlm.nih.gov/>

("Anti-Neutrophil Cytoplasmic Antibody-Associated Vasculitis"[mesh] OR "Antibodies, Antineutrophil Cytoplasmic"[mesh] OR "Systemic Vasculitis"[mesh] OR "Microscopic Polyangiitis"[mesh] OR "Churg-Strauss Syndrome"[mesh] OR "Granulomatosis with Polyangiitis"[mesh] OR "Glomerulonephritis"[mesh] OR "Nephritis"[mesh] OR "Vasculitis"[mesh] OR "Anti-Neutrophil Cytoplasmic Antibody-Associated Vasculitis"[tw] OR "Antineutrophil Cytoplasmic Antibodies"[tw] OR "Antineutrophil Cytoplasmic Antibody"[tw] OR "Systemic Vasculitis"[tw] OR "Microscopic Polyangiitis"[tw] OR "Churg-Strauss Syndrome"[tw] OR "Granulomatosis with Polyangiitis"[tw] OR "Glomerulonephritis"[tw] OR "Nephritis"[tw] OR "Vasculitis"[tw] OR "Vasculitides"[tw] OR "Angiitis"[tw] OR "Angiitides"[tw] OR "eosinophilic granulomatosis with polyangiitis"[tw] OR "Wegener's Granulomatosis"[tw] OR "wegener*"[tw] OR "Pulmonary renal syndrome"[tw] OR "Alveolar hemorrhag*"[tw] OR "Alveolar haemorrhag*"[tw] OR "Pulmonary hemorrhag*"[tw] OR "Pulmonary haemorrhag*"[tw] OR "Rapidly progressive glomerulonephritis with pulmonary hemorrhage"[Supplementary Concept] OR "Glomerulonephritis - pulmonary hemorrhage"[tw])

AND

("Plasma Exchange"[mesh] OR "Plasmapheresis"[mesh] OR "Plasma Exchange"[tw] OR "Plasmapheresis"[tw])

AND

("Randomized Controlled Trial"[Publication Type] OR "Randomized Controlled Trials as Topic"[Mesh] OR "Randomized"[tw] OR "Randomised"[tw] OR "RCT"[tw] OR "RCTs"[tw] OR "control groups"[mesh] OR "control group"[tw] OR "control groups"[tw] OR "controlled clinical trial"[pt] OR "controlled clinical trials as topic"[mesh] OR "cross-over studies"[mesh] OR "cross over study"[tw] OR "cross over studies"[tw] OR "double-blind method"[mesh] OR "double blind"[tw] OR "placebos"[mesh] OR placebo*[tw] OR placebos*[tw] OR "RaCT"[tw] OR "RaCTs"[tw] OR "random allocation"[mesh] OR "Research Design"[MeSH:noexp] OR "Research design"[tw] OR "Research designs"[tw] OR "single blind"[tw] OR "single-blind method"[mesh] OR ((single*[tw] OR double*[tw] OR triple*[tw]) AND (blind*[tw] OR mask*[tw])) OR volunteer*[tw] OR "trial"[ti] OR "Clinical Trial"[Publication Type])

**MEDLINE via OVID**

<http://gateway.ovid.com/ovidweb.cgi?T=JS&MODE=ovid&NEWS=n&PAGE=main&D=medall>

("Anti-Neutrophil Cytoplasmic Antibody-Associated Vasculitis"/ OR "Antibodies, Antineutrophil Cytoplasmic"/ OR "Systemic Vasculitis"/ OR "Microscopic Polyangiitis"/ OR "Churg-Strauss Syndrome"/ OR "Granulomatosis with Polyangiitis"/ OR "Glomerulonephritis"/ OR exp "Nephritis"/ OR exp "Vasculitis"/ OR "Anti-Neutrophil Cytoplasmic Antibody-Associated Vasculitis".mp OR "Antineutrophil Cytoplasmic Antibodies".mp OR "Antineutrophil Cytoplasmic Antibody".mp OR "Systemic Vasculitis".mp OR "Microscopic Polyangiitis".mp OR "Churg-Strauss Syndrome".mp OR "Granulomatosis with Polyangiitis".mp OR "Glomerulonephritis".mp OR "Nephritis".mp OR "Vasculitis".mp OR "eosinophilic granulomatosis with polyangiitis".mp OR "Wegener's Granulomatosis".mp OR "wegener*".mp OR "Pulmonary renal syndrome".mp OR "Alveolar hemorrhag*".mp OR "Alveolar haemorrhag*".mp OR "Pulmonary hemorrhag*".mp OR "Pulmonary haemorrhag*".mp OR "Rapidly progressive glomerulonephritis with pulmonary hemorrhage"/ OR "Glomerulonephritis - pulmonary hemorrhage".mp)

AND

("Plasma Exchange"/ OR exp "Plasmapheresis"/ OR "Plasma Exchange".mp OR "Plasmapheresis".mp)

AND

(exp "Randomized Controlled Trial"/ OR exp "Randomized Controlled Trials as Topic"/ OR "Randomized".mp OR "Randomised".mp OR "RCT".mp OR "RCTs".mp OR "control groups"/ OR "control group".mp OR "control groups".mp OR exp "controlled clinical trial"/ OR "controlled clinical trials as topic"/ OR "cross-over studies"/ OR "cross over study".mp OR "cross over studies".mp OR "double-blind method"/ OR "double blind".mp OR "placebos"/ OR placebo*.mp OR placebos*.mp OR "RaCT".mp OR "RaCTs".mp OR "random allocation"/ OR "Research Design"/ OR "Research design".mp OR "Research designs".mp OR "single blind".mp OR "single-blind method"/ OR ((single*.mp OR double*.mp OR triple*.mp) AND (blind*.mp OR mask*.mp)) OR volunteer*.mp OR "trial".ti OR exp "Clinical Trial"/)

**Embase**

<http://ovidsp.ovid.com/ovidweb.cgi?T=JS&PAGE=main&MODE=ovid&D=oemezd>

(*"ANCA associated vasculitis"/ OR *"Anti-Neutrophil Cytoplasmic Antibody-Associated Vasculitis"/ OR *"neutrophil cytoplasmic antibody"/ OR "*Systemic Vasculitis"/ OR *"Microscopic Polyangiitis"/ OR *"Churg-Strauss Syndrome"/ OR *"Granulomatosis with Polyangiitis"/ OR *"Glomerulonephritis"/ OR exp *"Nephritis"/ OR exp *"Vasculitis"/ OR "Anti-Neutrophil Cytoplasmic Antibody-Associated Vasculitis".ti,ab OR "Antineutrophil Cytoplasmic Antibodies".ti,ab OR "Antineutrophil Cytoplasmic Antibody".ti,ab OR "Systemic Vasculitis".ti,ab OR "Microscopic Polyangiitis".ti,ab OR "Churg-Strauss Syndrome".ti,ab OR "Granulomatosis with Polyangiitis".ti,ab OR "Glomerulonephritis".ti,ab OR "Nephritis".ti,ab OR "Vasculitis".ti,ab OR "eosinophilic granulomatosis with polyangiitis".ti,ab OR "Wegener's Granulomatosis".ti,ab OR "wegener*".ti,ab OR "Pulmonary renal syndrome".ti,ab OR "Alveolar hemorrhag*".ti,ab OR "Alveolar haemorrhag*".ti,ab OR "Pulmonary hemorrhag*".ti,ab OR "Pulmonary haemorrhag*".ti,ab OR "Rapidly progressive glomerulonephritis with pulmonary hemorrhage".ti,ab OR "Glomerulonephritis - pulmonary hemorrhage".ti,ab)

AND

(*"Plasma Exchange"/ OR exp *"Plasmapheresis"/ OR "Plasma Exchange".ti,ab OR "Plasmapheresis".ti,ab)

AND

(exp "Randomized Controlled Trial"/ OR exp "Randomized Controlled Trials as Topic"/ OR "Randomized".ti,ab OR "Randomised".ti,ab OR "RCT".ti,ab OR "RCTs".ti,ab OR "control groups"/ OR "control group".ti,ab OR "control groups".ti,ab OR exp "controlled clinical trial"/ OR "controlled clinical trials as topic"/ OR "cross-over studies"/ OR "cross over study".ti,ab OR "cross over studies".ti,ab OR "double-blind method"/ OR "double blind".ti,ab OR "placebos"/ OR placebo*.ti,ab OR placebos*.ti,ab OR "RaCT".ti,ab OR "RaCTs".ti,ab OR "random allocation"/ OR "Research Design"/ OR "Research design".ti,ab OR "Research designs".ti,ab OR "single blind".ti,ab OR "single-blind method"/ OR ((single*.ti,ab OR double*.ti,ab OR triple*.ti,ab) AND (blind*.ti,ab OR mask*.ti,ab)) OR volunteer*.ti,ab OR "trial".ti OR exp "Clinical Trial"/)

**Cochrane**

<https://www.cochranelibrary.com/advanced-search/search-manager>

("Anti-Neutrophil Cytoplasmic Antibody-Associated Vasculitis" OR "Antibodies, Antineutrophil Cytoplasmic" OR "Systemic Vasculitis" OR "Microscopic Polyangiitis" OR "Churg-Strauss Syndrome" OR "Granulomatosis with Polyangiitis" OR "Glomerulonephritis" OR "Nephritis" OR "Vasculitis" OR "Anti-Neutrophil Cytoplasmic Antibody-Associated Vasculitis" OR "Antineutrophil Cytoplasmic Antibodies" OR "Antineutrophil Cytoplasmic Antibody" OR "Systemic Vasculitis" OR "Microscopic Polyangiitis" OR "Churg-Strauss Syndrome" OR "Granulomatosis with Polyangiitis" OR "Glomerulonephritis" OR "Nephritis" OR "Vasculitis" OR "eosinophilic granulomatosis with polyangiitis" OR "Wegener's Granulomatosis" OR "wegener*" OR "Pulmonary renal syndrome" OR "Alveolar hemorrhag*" OR "Alveolar haemorrhag*" OR "Pulmonary hemorrhag*" OR "Pulmonary haemorrhag*" OR "Rapidly progressive glomerulonephritis with pulmonary hemorrhage" OR "Glomerulonephritis pulmonary hemorrhage"):ti,ab,kw

AND

("Plasma Exchange" OR "Plasmapheresis" OR "Plasma Exchange" OR "Plasmapheresis"):ti,ab,kw
