# Supplementary material for: Efficacy of plasma exchange for antineutrophil cytoplasmic antibody-associated systemic vasculitis: a systematic review and meta-analysis
Source: Arthritis Res Ther. 2021 Jan 14;23:28. doi: 10.1186/s13075-021-02415-z (PMC7809754; doi:10.1186/s13075-021-02415-z)
Supplement: Supplementary file 4 — Additional file 4. The result of the 39 full-text records assessed for eligibility. [file 13075_2021_2415_MOESM4_ESM.docx]

**Additional file 4. The result of the 39 full text records assessed for eligibility.**

1. Casian, A. Plasma exchange for severe renal vasculitis: Long-term follow-up of the MEPEX trial. *Clinical and Experimental Immunology* **164**, 52, doi:http://dx.doi.org/10.1111/j.1365-2249.2011.04337.x (2011).

**Include**

1. Cohen, J. Plasma exchange in glomerulonephritis. *Kidney* **14**, 41-45 (1981).

**Exclude Reason: Review.**

3 Cole, E. *et al.* A prospective randomized trial of plasma exchange as additive therapy in idiopathic crescentic glomerulonephritis. The Canadian Apheresis Study Group. *American journal of kidney diseases* **20**, 261‐269, doi:10.1016/s0272-6386(12)80699-8 (1992).

**Exclude Reason: Not randomized controlled trial (RCT)**

4 Danieli, M. G. *et al.* Long term effectiveness of intravenous immunoglobulin in Churg-Strauss syndrome. *Ann Rheum Dis* **63**, 1649-1654, doi:10.1136/ard.2003.015453 (2004).

**Include**

5 Gaskin, G. & Jayne, D. Adjunctive plasma exchange is superior to methylprednisolone in acute renal failure due to ANCA-associated glomerulonephritis. *Journal of the american society of nephrology : JASN* **13**, 2A (2002).

**Exclude Reason: Only 18 of 78 (23%) participants were Churg-Strauss syndrome (CSS). 60 of 78 (77%) were diagnosed as polyarteritis nodosa (PAN).**

6 Glöckner, W. M. *et al.* Plasma exchange and immunosuppression in rapidly progressive glomerulonephritis: a controlled, multi-center study. *Clinical nephrology* **29**, 1‐8 (1988).

**Exclude Reason: No diagnosis of ANCA-associated vasculitis (AAV). Rapidly progressive glomerulonephritis (RPGN) caused by other disease such as Lupus may be included.**

7 Guillevin, L. *et al.* Lack of superiority of steroids plus plasma exchange to steroids alone in the treatment of polyarteritis nodosa and Churg-Strauss syndrome: A prospective, randomized trial in 78 patients. *Arthritis and Rheumatism* **35**, 208-215 (1992).

**Exclude Reason: Only 18 of 78 (23%) were CSS. 60 of 78 (77%) were diagnosed as PAN.**

8 Guillevin, L. *et al.* Longterm followup after treatment of polyarteritis nodosa and Churg-Strauss angiitis with comparison of steroids, plasma exchange and cyclophosphamide to steroids and plasma exchange. A prospective randomized trial of 71 patients. The Cooperative Study Group for Polyarteritis Nodosa. *J Rheumatol* **18**, 567-574 (1991).

**Excluded Reason All participants received plasma exchange (PE).**

9 Guillevin, L. *et al.* Corticosteroids plus pulse cyclophosphamide and plasma exchanges versus corticosteroids plus pulse cyclophosphamide alone in the treatment of polyarteritis nodosa and Churg-Strauss syndrome patients with factors predicting poor prognosis: A prospective, randomized trial in sixty-two patients. *Arthritis and Rheumatism* **38**, 1638-1645, doi:http://dx.doi.org/10.1002/art.1780381116 (1995).

**Excluded Reason Only 14 of 62 (23%) were CSS. 48 of 62 (77%) were diagnosed as PAN.**

10 Jayne, D. R. *et al.* Randomized trial of plasma exchange or high-dosage methylprednisolone as adjunctive therapy for severe renal vasculitis. *Journal of the american society of nephrology : JASN* **18**, 2180‐2188, doi:10.1681/asn.2007010090 (2007).

**Include**

11 Keller, E., Beeser, H., Peter, H. H., Arnold, A. & Kotitschke, R. Comparison of fresh frozen plasma with a standardized serum protein solution following therapeutic plasma exchange in patients with autoimmune disease: a prospective controlled clinical trial. *Therapeutic apheresis* **4**, 332‐337, doi:10.1046/j.1526-0968.2000.004005332.x (2000).

**Excluded Reason: Only 3 of 24 (13%) were AAV.**

12 Kerr, P. G., Chadban, S. J. & Atkins, R. C. Is there a role for plasma exchange in rapidly progressive glomerulonephritis? *Nephrology* **6**, 141-143, doi:http://dx.doi.org/10.1046/j.1440-1797.2001.00052.x (2001).

**Exclude Reason: Review**

13 Lhote, F. *et al.* Side effects of therapeutic plasma exchange during treatment of polyarteritis nodosa. Comparison of filtration and centrifugation. 718 sessions in 63 patients. *Life Support Syst* **5**, 359-366 (1987).

**Exclude Reason: All participants received PE.**

14 Lhote, F. *et al.* Complications of plasma exchange in the treatment of polyarteritis nodosa and Churg-Strauss angiitis and the contribution of adjuvant immunosuppressive therapy: a randomized trial in 72 patients. *Artif Organs* **12**, 27-33, doi:10.1111/j.1525-1594.1988.tb01520.x (1988).

**Exclude Reason: All participants received PE.**

15 Mauri, J. M., Gonzalez, M. T. & Poveda, R. Therapeutic plasma exchange in the treatment of rapidly progressive glomerulonephritis. *Plasma Therapy and Transfusion Technology* **6**, 587-591 (1985).

**Exclude Reason: Only 3 (Wegener's granulomatosis [WG]) of 22 (14%) were diagnosed as AAV.**

16 Metz-Kurschel, U., Graben, N. & Daul, A. Rapidly progressing glomerulonephritis. Spontaneous course and differential therapy with special reference to the infection-associated form. *Klinische Wochenschrift* **67**, 621‐626, doi:10.1007/bf01718143 (1989).

**Exclude. Reason: Only 7 of 31 (23%) were AAV (WG). Not RCT.**

17 Nct. Plasma Exchange for Renal Vasculitis. *https://clinicaltrials.gov/show/NCT01408836* (2011).

**Include**

18 Nct. The Clinical Efficacy of DFPP in Patients With AAGN. *https://clinicaltrials.gov/show/NCT02294344* (2014).

**Include (Terminated. This was because the recruitment of subject was very difficult.)**

19 Nct. Plasma Exchange and Glucocorticoids for Treatment of Anti-Neutrophil Cytoplasm Antibody (ANCA) - Associated Vasculitis (PEXIVAS) - Glucocorticoids. *https://clinicaltrials.gov/show/NCT03919825* (2019).

**Include**

20 Pusey, C. D., Rees, A. J., Evans, D. J., Peters, D. K. & Lockwood, C. M. Plasma exchange in focal necrotizing glomerulonephritis without anti-GBM antibodies. *Kidney Int* **40**, 757-763, doi:10.1038/ki.1991.272 (1991).

**Include (Idiopathic RPGN is mixed, but more than 75% of patients are diagnosed with microscopic polyangiitis [MPA] or Granulomatosis with polyangiitis [GPA].)**

21 Rees, A. J. & Pusey, C. D. Plasma exchange in systemic vasculitis. *Netherlands Journal of Medicine* **36**, 103-106 (1990).

**Exclude Reason: Review.**

22 Rifle, G. *et al.* Treatment of idiopathic acute crescentic glomerulonephritis by immunodepression and plasma-exchanges. A prospective randomised study. *Proc Eur Dial Transplant Assoc* **18**, 493-502 (1981).

**Exclude Reason: No diagnosis of AAV.**

23 Rifle, G. & Dechelette, E. Treatment of rapidly progressive glomerulonephritis by plasma exchange and methylprednisolone pulses. A prospective randomized trial of cyclophosphamide. Interim analysis. The French Cooperative Group. *Prog Clin Biol Res* **337**, 263-267 (1990).

**Exclude Reason: No diagnosis of AAV.**

24 Salant, D. J. Intravenous methylprednisolone or plasma exchange for adjunctive therapy of severe renal vasculitis? *Nature Clinical Practice Nephrology* **4**, 14-15, doi:http://dx.doi.org/10.1038/ncpneph0650 (2008).

**Include**

25 Samson, M. *et al.* Long-term follow-up of a randomized trial on 118 patients with polyarteritis nodosa or microscopic polyangiitis without poor-prognosis factors. *Autoimmunity reviews* **13**, 197‐205, doi:10.1016/j.autrev.2013.10.001 (2014).

**Exclude Reason: Comparison of azathioprine with cyclophosphamide.**

26 Sanz-Guajardo, D. Plasmapheresis in the treatment of glomerulonephritis: indications and complications. *American journal of kidney diseases : the official journal of the National Kidney Foundation* **36**, LIV-VI (2000).

**Exclude Reason: Review**

27 Smith, J. W., Abe, Y., Blasutig, E. & Nose, Y. Use of cryofiltration and plasma exchange in the treatment of systemic vasculitis. *Transactions - American Society for Artificial Internal Organs* **30**, 179-183 (1984).

**Exclude Reason: Not RCT. (a case series)**

28 Stegmayr, B. G. *et al.* Plasma exchange or immunoadsorption in patients with rapidly progressive crescentic glomerulonephritis. A Swedish multi-center study. *International journal of artificial organs* **22**, 81‐87 (1999).

**Exclude Reason: Eleven of 44 (25%) were not diagnosed as AAV. (6 of the 11 were Goodpasture’s syndrome.)**

29 Stegmayr, B. G. *et al.* Plasma exchange versus immunoadsorption in the treatment of rapidly progressive glomerulonephritis (rpg). A multi-center study. *35th congress. European renal association. European dialysis and transplantation association; 1998 jun 6-9; rimini, italy*, 294 (1998).

**Exclude Reason: The same as 27.**

30 Szpirt, W. M., Heaf, J. G. & Petersen, J. Plasma exchange for induction and cyclosporine A for maintenance of remission in Wegener's granulomatosis--a clinical randomized controlled trial. *Nephrol Dial Transplant* **26**, 206-213, doi:10.1093/ndt/gfq360 (2011).

**Include**

31 Szpirt, W. M., Rasmussen, N. & Petersen, J. Plasma exchange for induction and cyclosporin A for maintenance of remission in Wegener's granulomatosis. *Journal of the american society of nephrology : JASN* **7**, 1781 (1996).

**Include**

32 Szpirt, W. M., Rasmussen, N. & Petersen, J. Long term outcome and prognostic factors in randomized study of plasma exchange and cyclosporin-A in Wegener's granulomatosis. *Journal of the american society of nephrology : JASN* **10**, 182A (1999).

**Include**

33 Walsh, M. *et al.* Long-term follow-up of patients with severe ANCA-associated vasculitis comparing plasma exchange to intravenous methylprednisolone treatment is unclear. *Kidney Int* **84**, 397-402, doi:10.1038/ki.2013.131 (2013).

**Include**

34 Walsh, M., Merkel, P. & Jayne, D. The effect of plasma exchange on end-stage renal disease and death in patients with severe ANCA-associated vasculitis. *Rheumatology* **58**, doi:http://dx.doi.org/10.1093/rheumatology/kez063.084 (2019).

**Include**

35 Walsh, M. *et al.* Plasma exchange and glucocorticoid dosing in the treatment of anti-neutrophil cytoplasm antibody associated vasculitis: Baseline characteristics of a randomized controlled trial (PEXIVAS). *Rheumatology (United Kingdom)* **56 (Supplement 3)**, iii149, doi:http://dx.doi.org/10.1093/rheumatology/kex134 (2017).

**Include**

36 Walsh, M., Merkel, P. A. & Jayne, D. The effects of plasma exchange and reduced-dose glucocorticoids during remission-induction for treatment of severe ANCA-associated vasculitis. *Arthritis and rheumatology* **70**, 3136‐, doi:10.1002/art.40700 (2018).

**Include**

37 Walsh, M. *et al.* Plasma exchange and glucocorticoid dosing in the treatment of anti-neutrophil cytoplasm antibody associated vasculitis (PEXIVAS): protocol for a randomized controlled trial. *Trials* **14**, 73, doi:10.1186/1745-6215-14-73 (2013).

**Include**

38 Walsh, M. *et al.* Plasma Exchange and Glucocorticoids in Severe ANCA-Associated Vasculitis. *New England Journal of Medicine* **382**, 622-631, doi:https://dx.doi.org/10.1056/NEJMoa1803537 (2020).

**Include (Although the eligibility criteria was set as 15 years of age or older, the mean age minus 2SD of actual cohort was over 18. Therefore, more than 80% of the participants were judged to be 18 years of age or older.)**

39 Zauner, I. *et al.* Predictive value of initial histology and effect of plasmapheresis on long-term prognosis of rapidly progressive glomerulonephritis. *American journal of kidney diseases* **39**, 28‐35 (2002).

**Include　(Type II RPGN was mixed, but more than 80% of patients were diagnosed with MPA or GPA.)**
