# Supplementary material for: Efficacy of plasma exchange for antineutrophil cytoplasmic antibody-associated systemic vasculitis: a systematic review and meta-analysis
Source: Arthritis Res Ther. 2021 Jan 14;23:28. doi: 10.1186/s13075-021-02415-z (PMC7809754; doi:10.1186/s13075-021-02415-z)
Supplement: Supplementary file 5 — Additional file 5. Supplementary tables and figures. [file 13075_2021_2415_MOESM5_ESM.docx]

**Additional file 5. Supplementary tables and figures.**

Supplementary table S1. Supplementary summary of included RCTs in this review

| Characteristics | PEXIVAS 2020 | Szpirt 2011 | Zäuner 2002 | Pusey 1991 | MEPEX 2007, 2013 |
| --- | --- | --- | --- | --- | --- |
| Observational period | Max 7 years (median 2.9 years) | Max 5 years (median 4.6 years) | Max 10 years (weighted average 2.0 years) | Max 11 years (median 4 years) | Max 10 years (median 3.95 years) |
| ***ANCA status*** |  |  |  |  |  |
| PR3-ANCA(C-ANCA) | 41% | 100% | 69% | N/D | 43% |
| MPO-ANCA(P-ANCA) | 59% | N/D | 18% | N/D | 52% |
| ***Organ involvement†*** |  |  |  |  |  |
| Kidney | 98% | 69%* | 100% | 100% | 100% |
| Lung | 42% | 66% | N/D | N/D | N/D |
| Nervous | 9% | 9% | N/D | N/D | N/D |
| Skin | 11% | 22% | N/D | N/D | N/D |
| Ear, nose, and throat | 28% | 81% | N/D | N/D | N/D |
| ***Combined treatments*‡** |  |  |  |  |  |
| GC | i.v. and p.o. | p.o. | i.v. and p.o. | p.o. | p.o. |
| CYC | i.v. or p.o. (if physician selected) | p.o. | p.o. | p.o. | p.o. |
| Rituximab | i.v. (if physician selected) | N/D | N/D | N/D | N/D |
| AZA | p.o. (after CYC treatment) | p.o. (if disease is clinically active after 12 months) | N/D | p.o. | p.o. |
| CyA | N/D | p.o. (if allocated to CyA group) | N/D | N/D | N/D |
| Chlorambuchil | N/D | p.o. (if AZA intolerability) | N/D | N/D | N/D |

†, partially omitted; * percentage of patients with elevated serum creatinine level, ‡other than intervention and control. Abbreviation; GC, glucocorticoid; CYC, cyclophosphamide; AZA, azathioprine; CyA, cyclosporine A; i.v., intravenous injection; p.o., per os (oral administration), N/D, not described


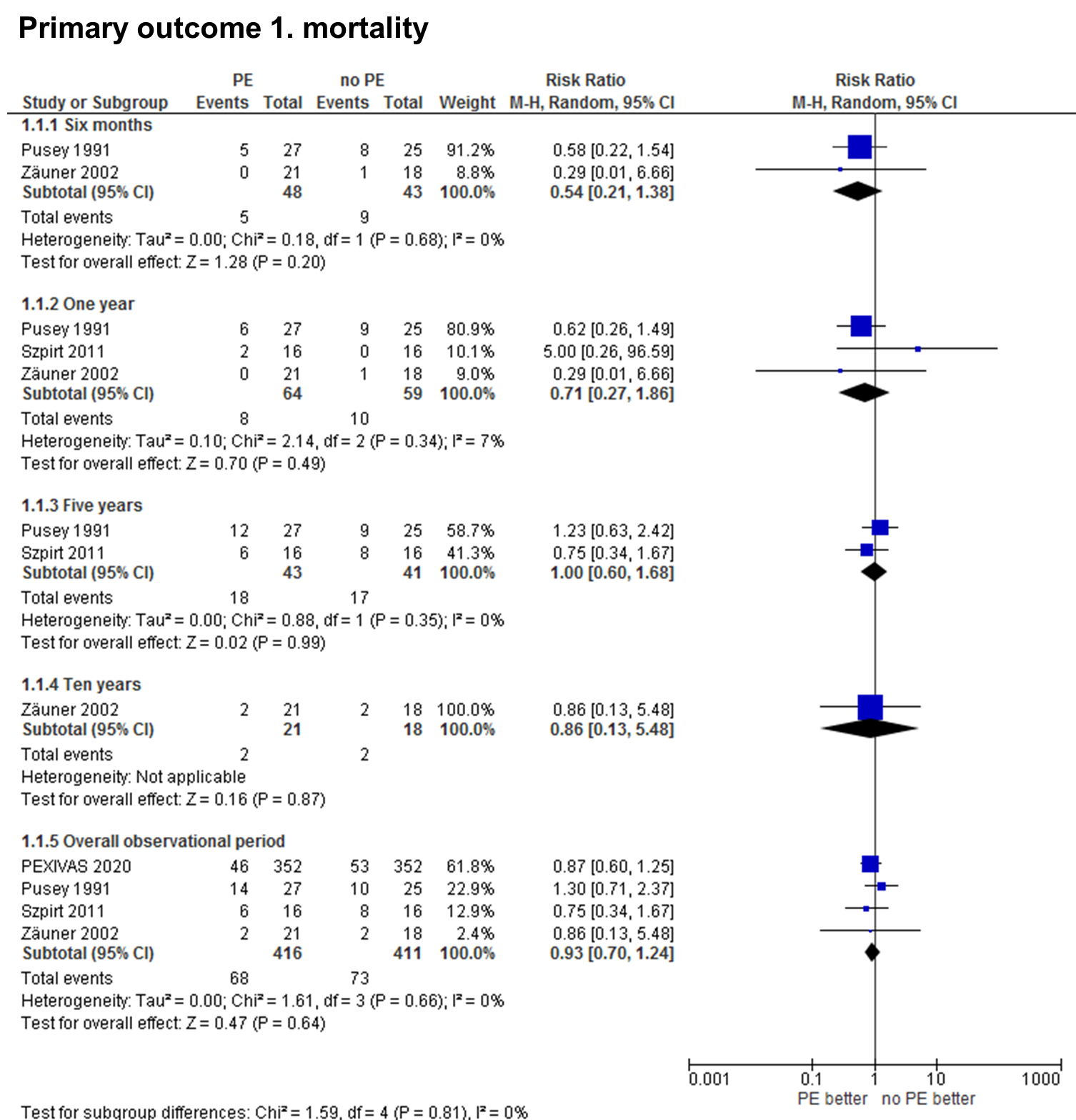


**Supplementary figure S1. Forest plot of mortality in patients with plasma exchange (PE) or no plasma exchange.**

**
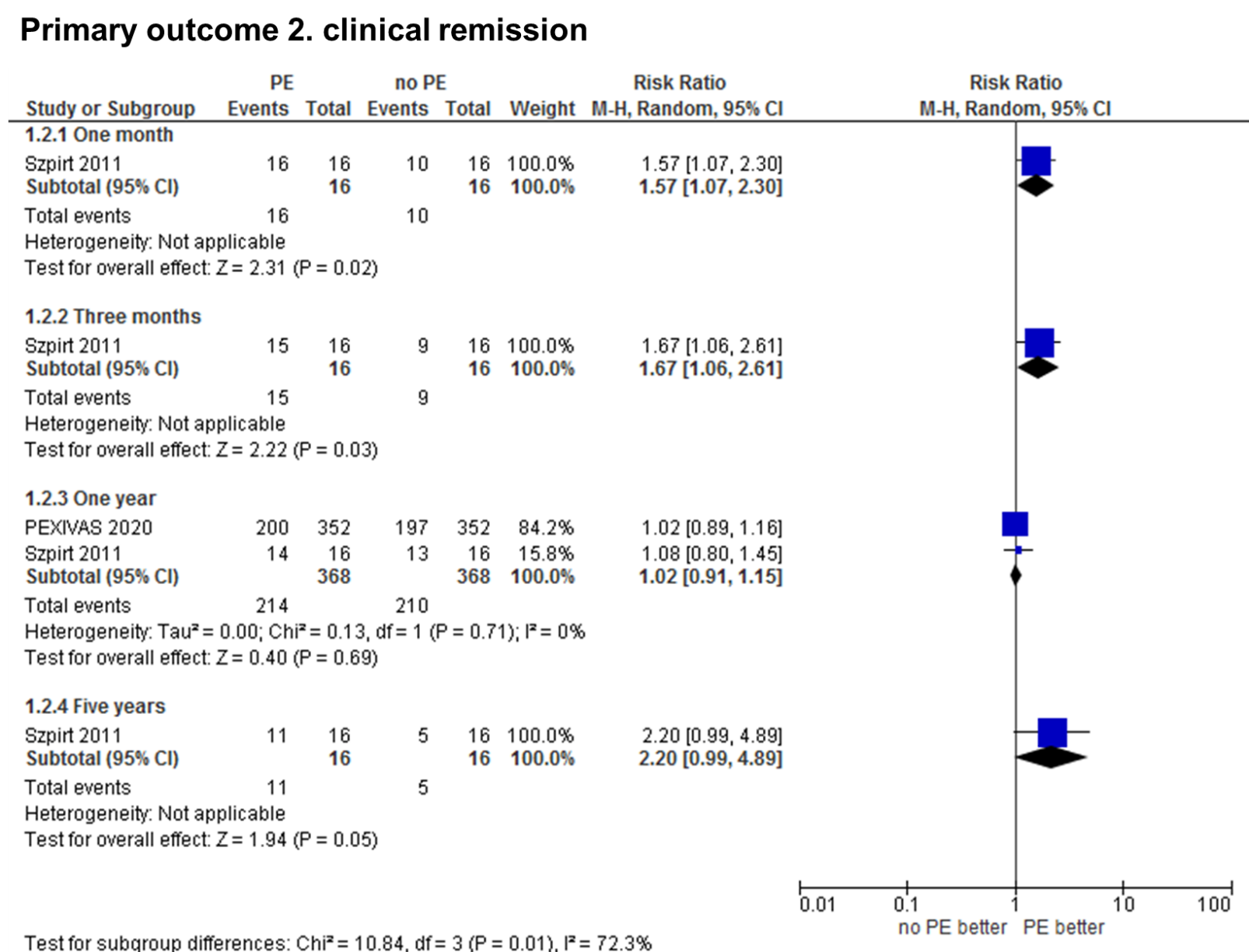
**

**Supplementary figure S2. Forest plot of clinical remission in patients with plasma exchange (PE) or no plasma exchange.**

**
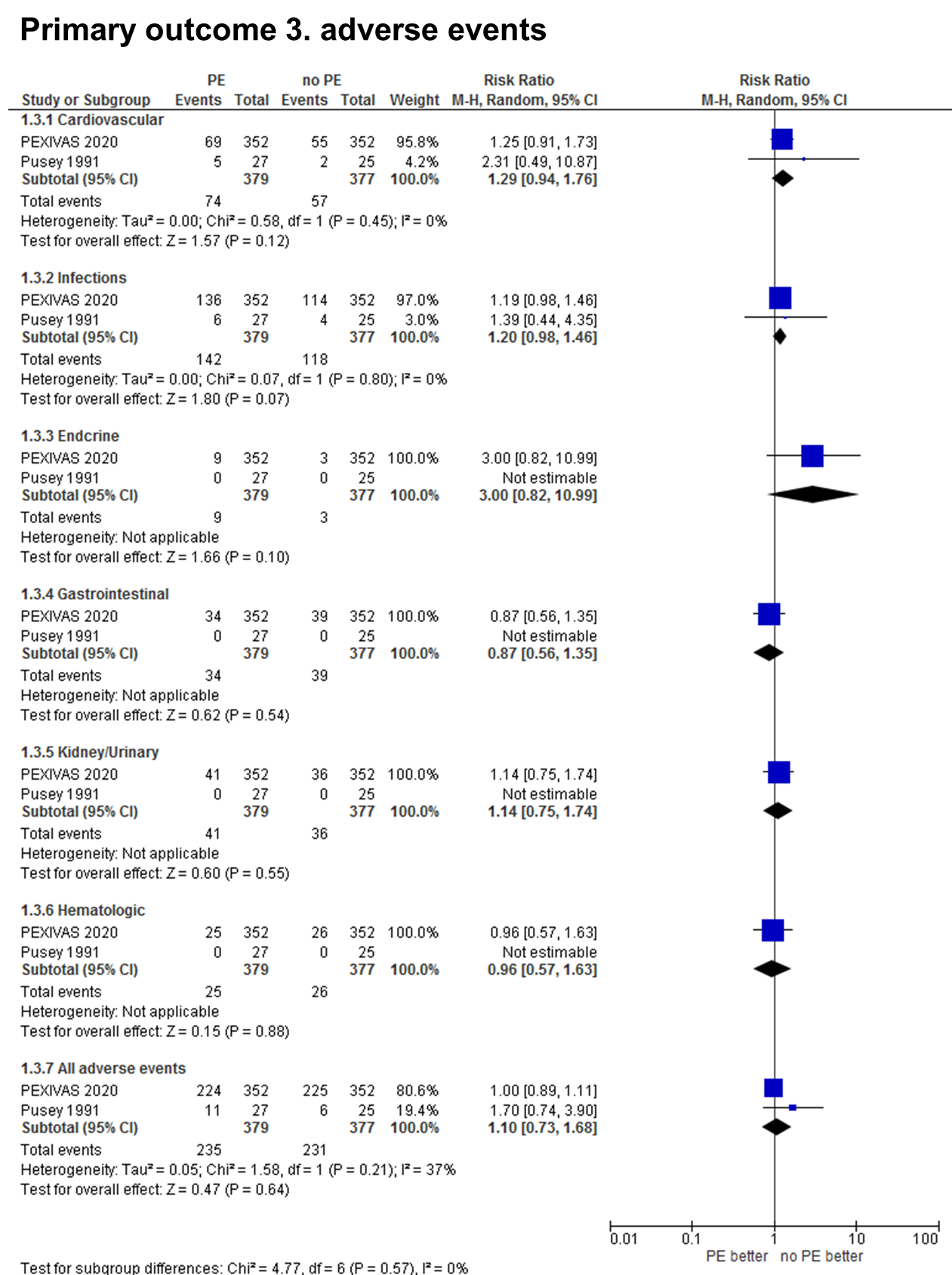
**

**Supplementary figure S3. Forest plot of adverse events in patients with plasma exchange (PE) or no plasma exchange.**

**
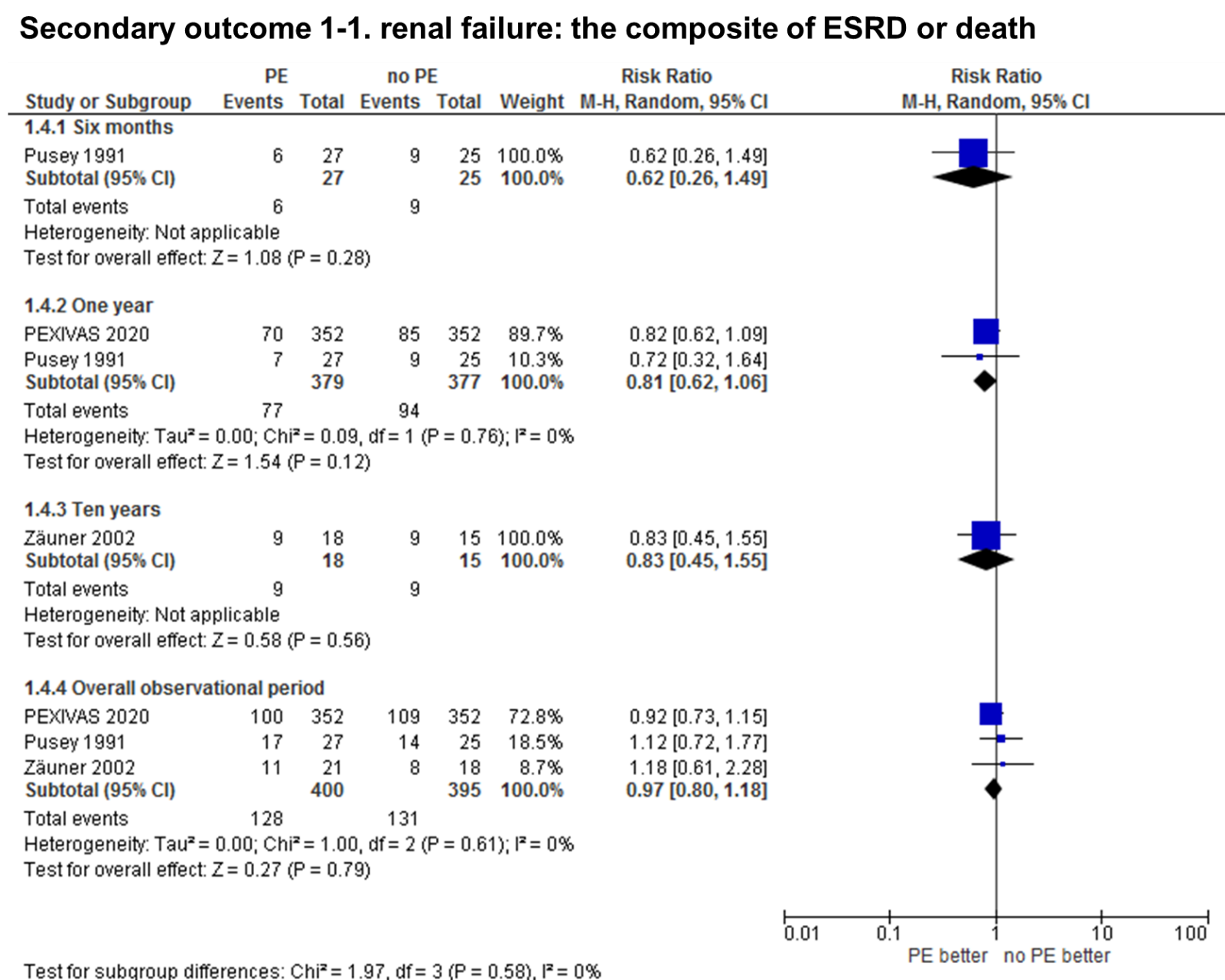
**

**Supplementary figure S4. Forest plot of the composite of end-stage renal disease (ESRD) in patients with plasma exchange (PE) or no plasma exchange.**

**
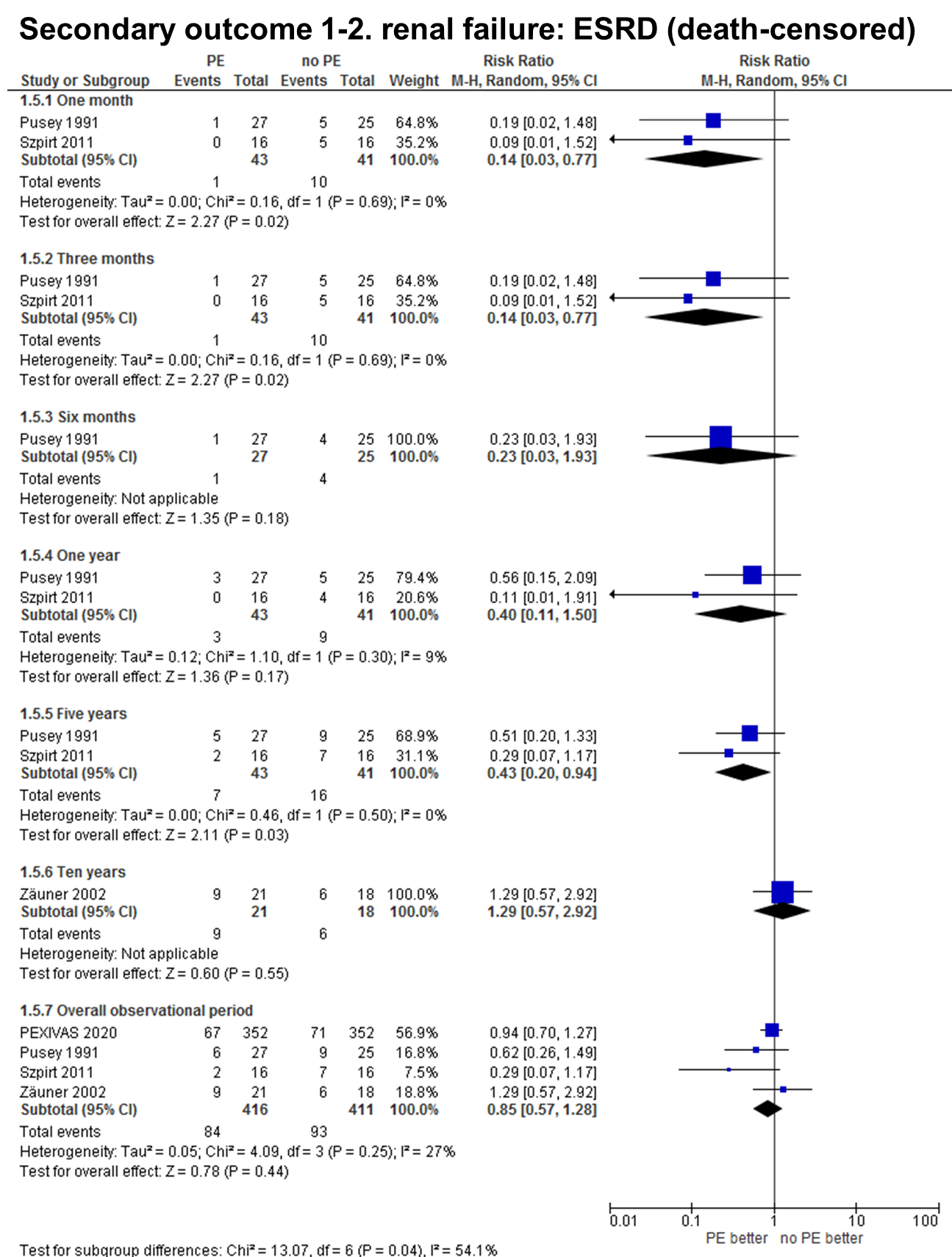
**

**Supplementary figure S5. Forest plot of end-stage renal disease (ESRD) (death-censored) in patients with plasma exchange (PE) or no plasma exchange.**

**
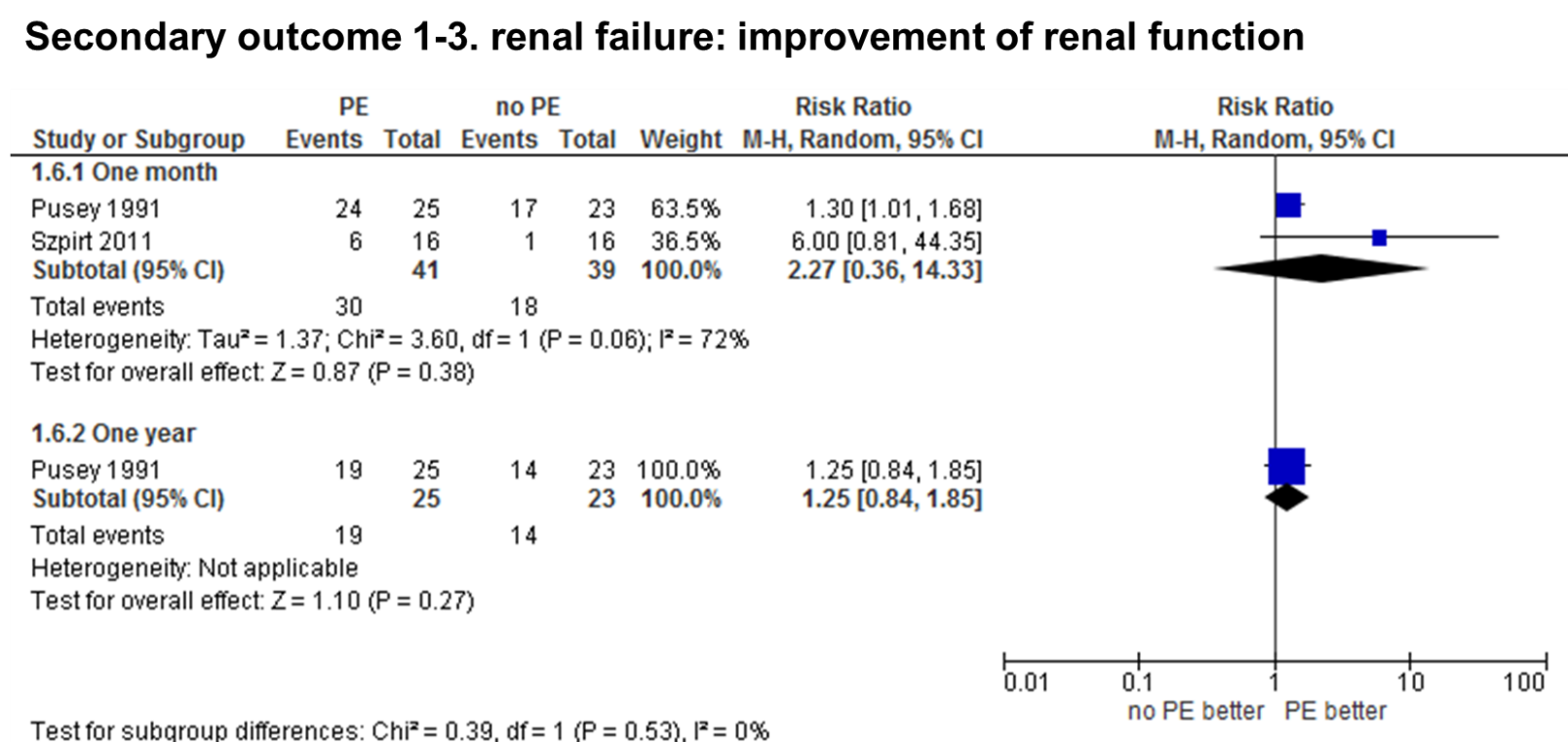
**

**Supplementary figure S6. Forest plot of improvement of renal function in patients with plasma exchange (PE) or no plasma exchange.**

**
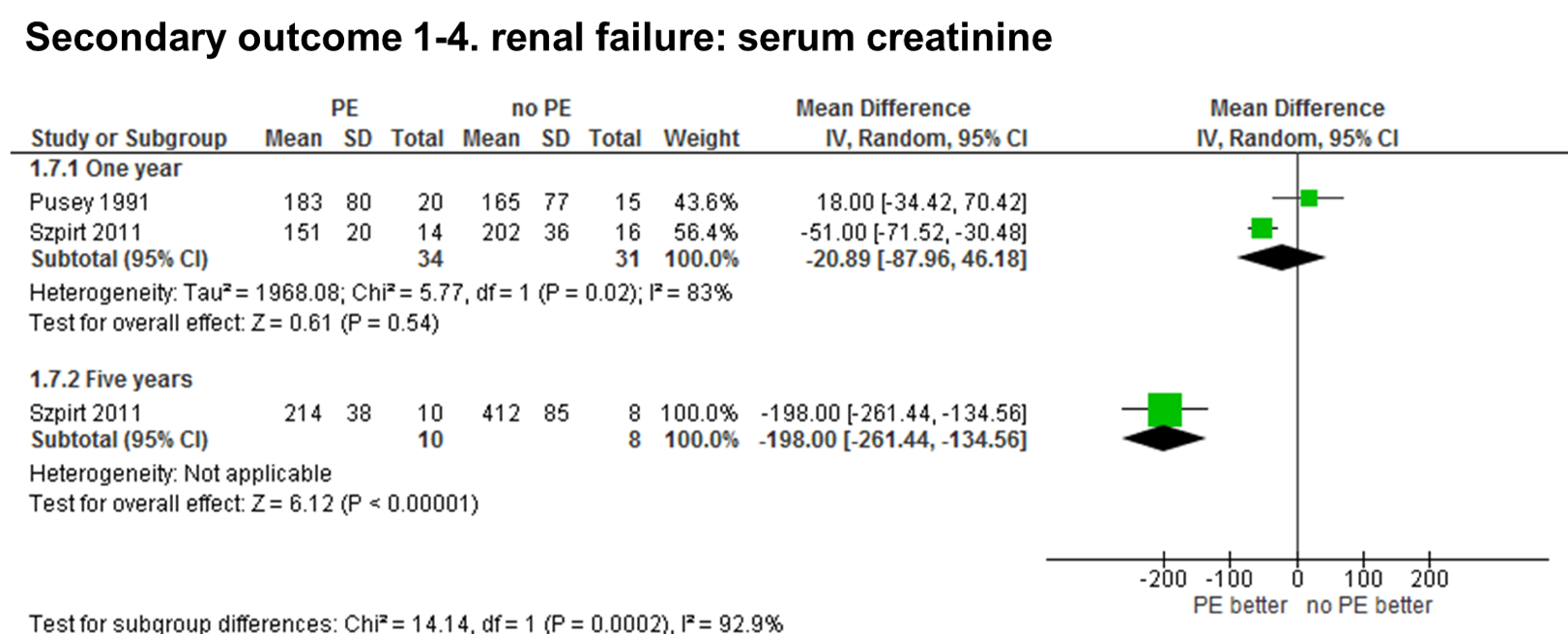
**

**Supplementary figure S7. Forest plot of serum creatinine in patients with plasma exchange (PE) or no plasma exchange.** Unit, µmol/L

**
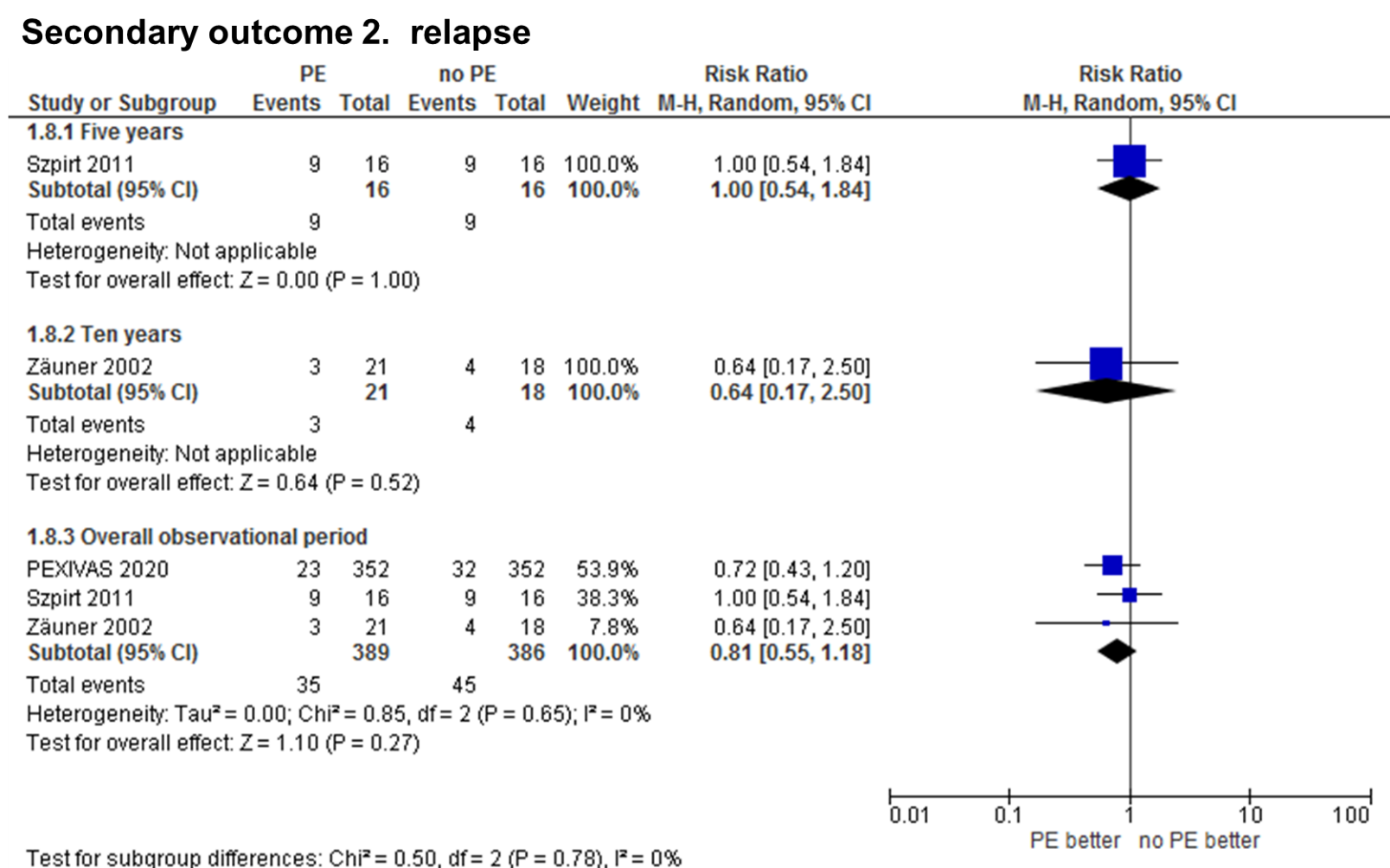
**

**Supplementary figure S8. Forest plot of relapse in patients with plasma exchange (PE) or no plasma exchange.**

**
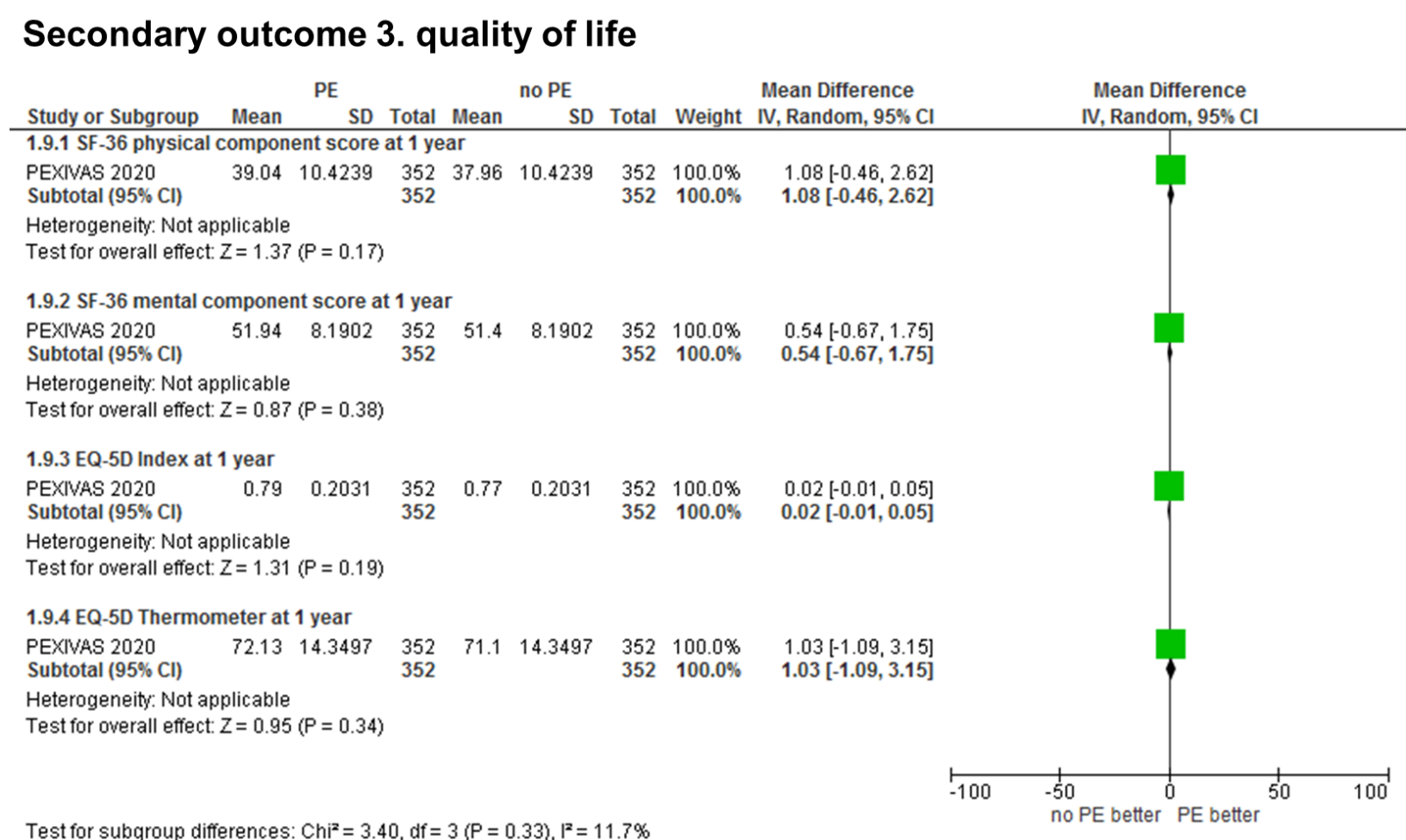
**

**Supplementary figure S9. Forest plot of quality of life in patients with plasma exchange (PE) or no plasma exchange.**

**
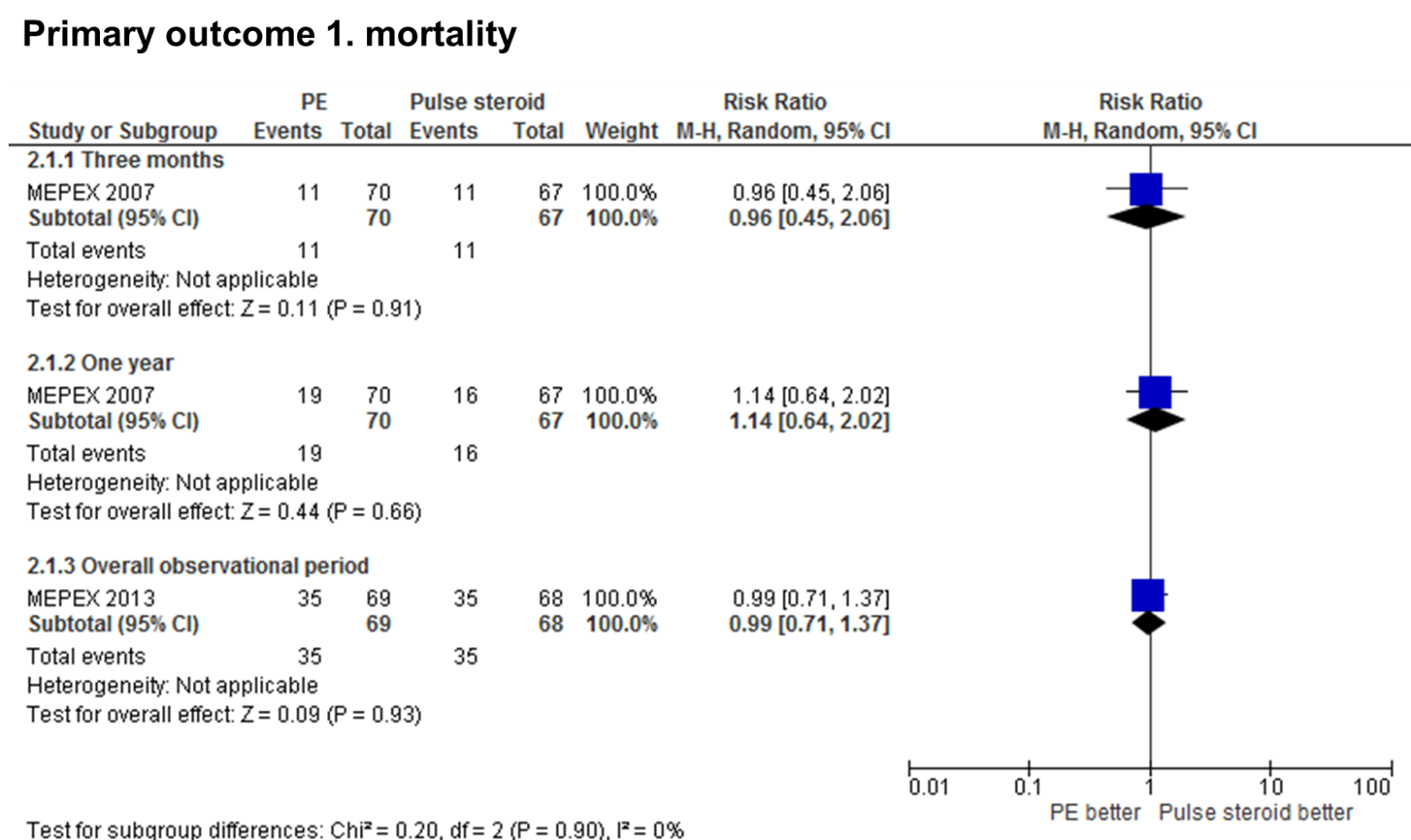
**

**Supplementary figure S10. Forest plot of mortality in patients with plasma exchange (PE) or pulse steroid treatment.**

**
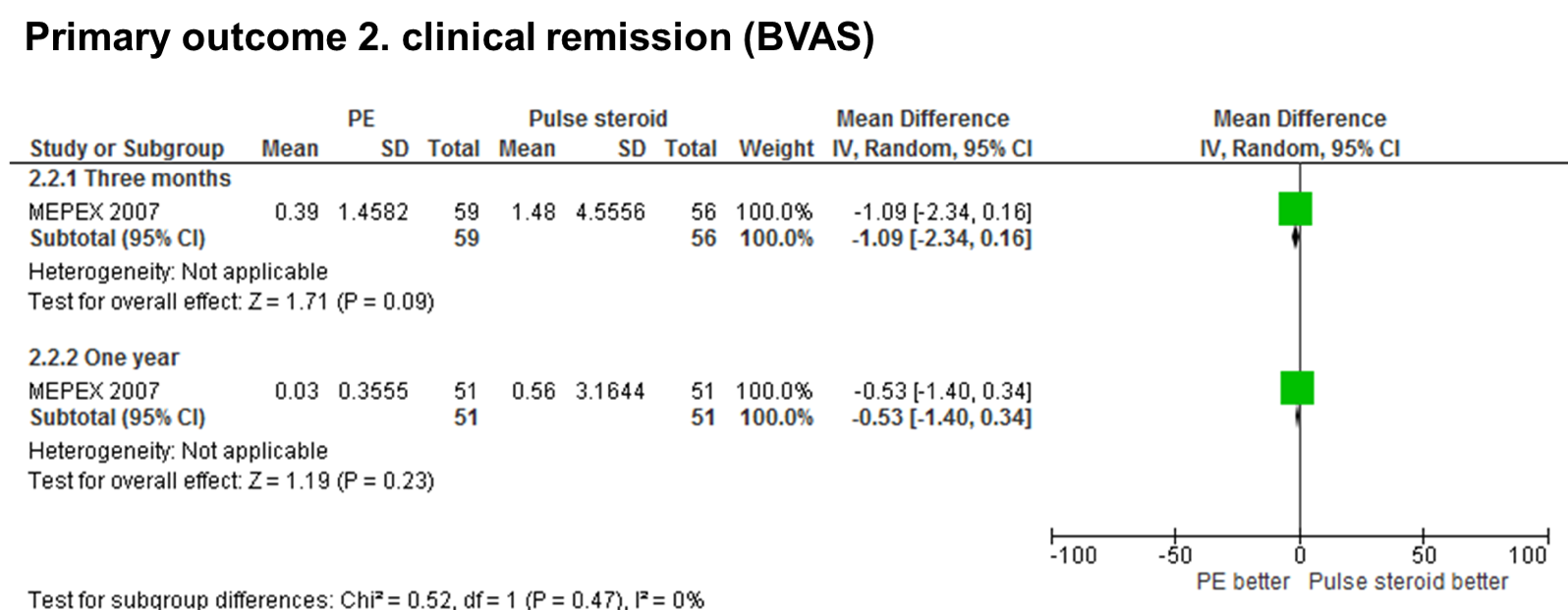
**

**Supplementary figure S11. Forest plot of Birmingham Vasculitis Activity Score (BVAS) in patients with plasma exchange (PE) or pulse steroid treatment.** BVAS was compared as continuous value.

**
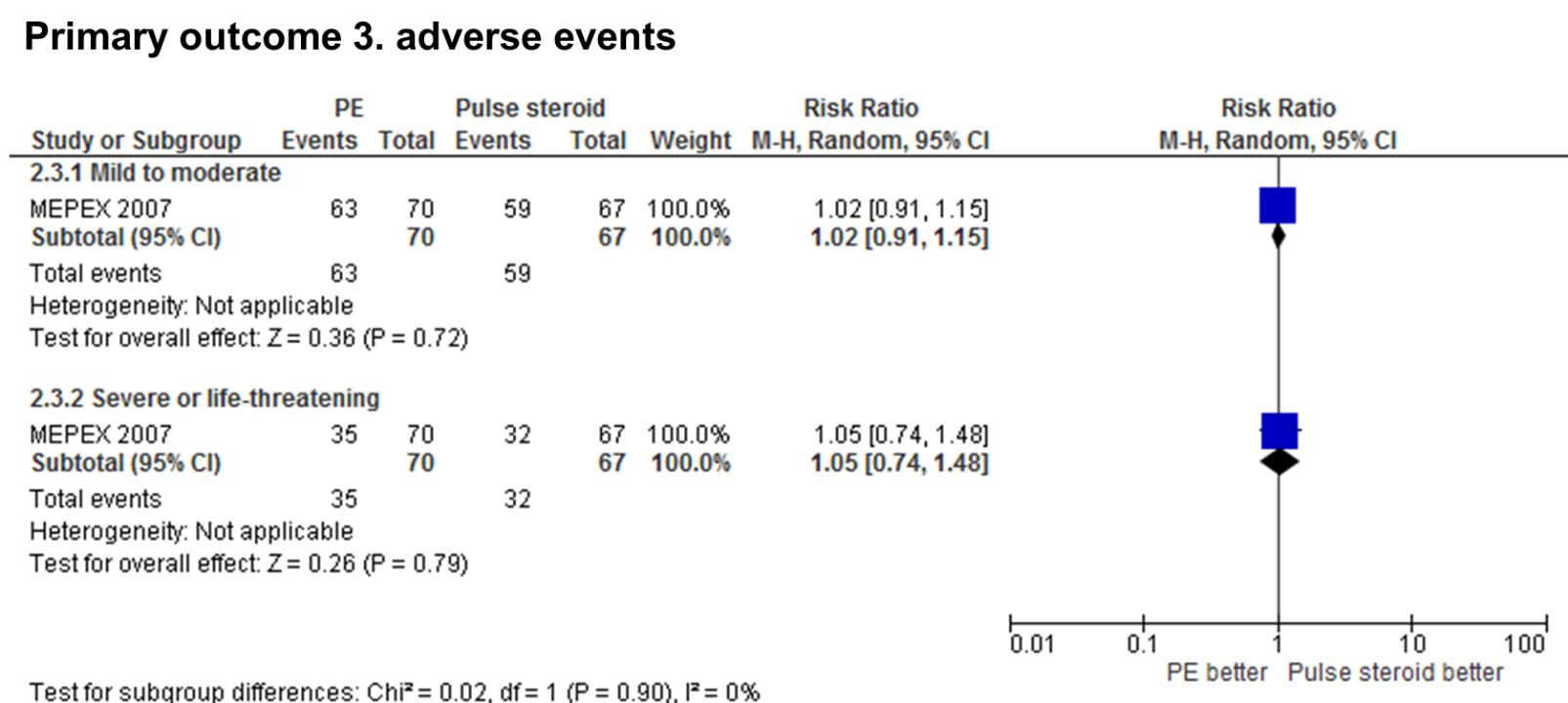
**

**Supplementary figure S12. Forest plot of adverse events in patients with plasma exchange (PE) or pulse steroid treatment.**

**
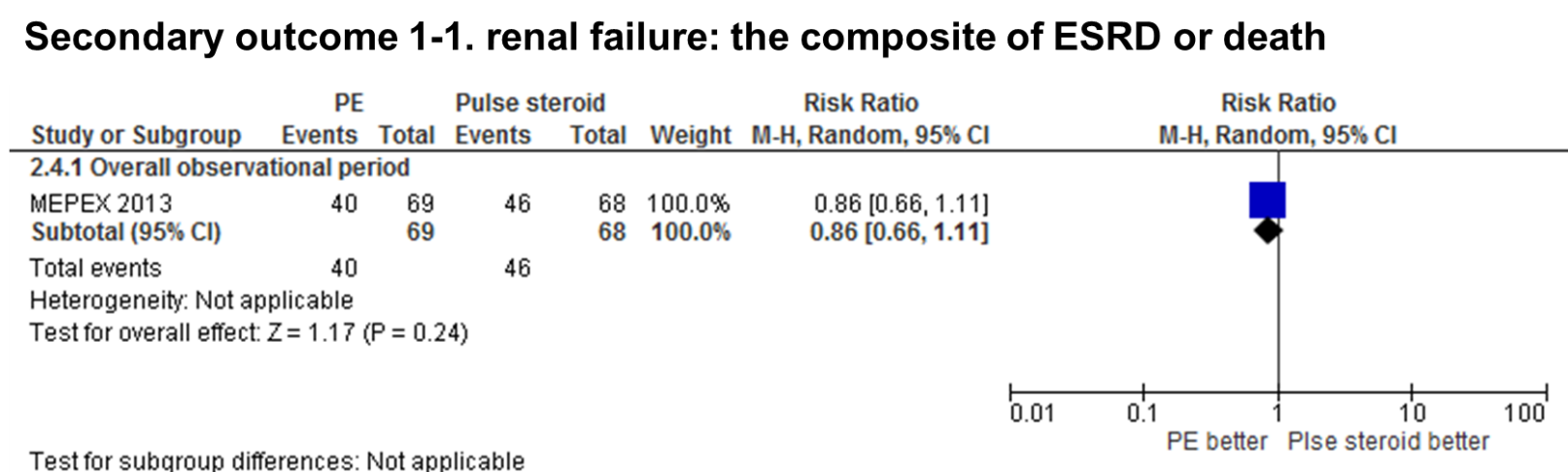
**

**Supplementary figure S13. Forest plot of the composite of end-stage renal disease (ESRD) or death in patients with plasma exchange (PE) or pulse steroid treatment.**

**
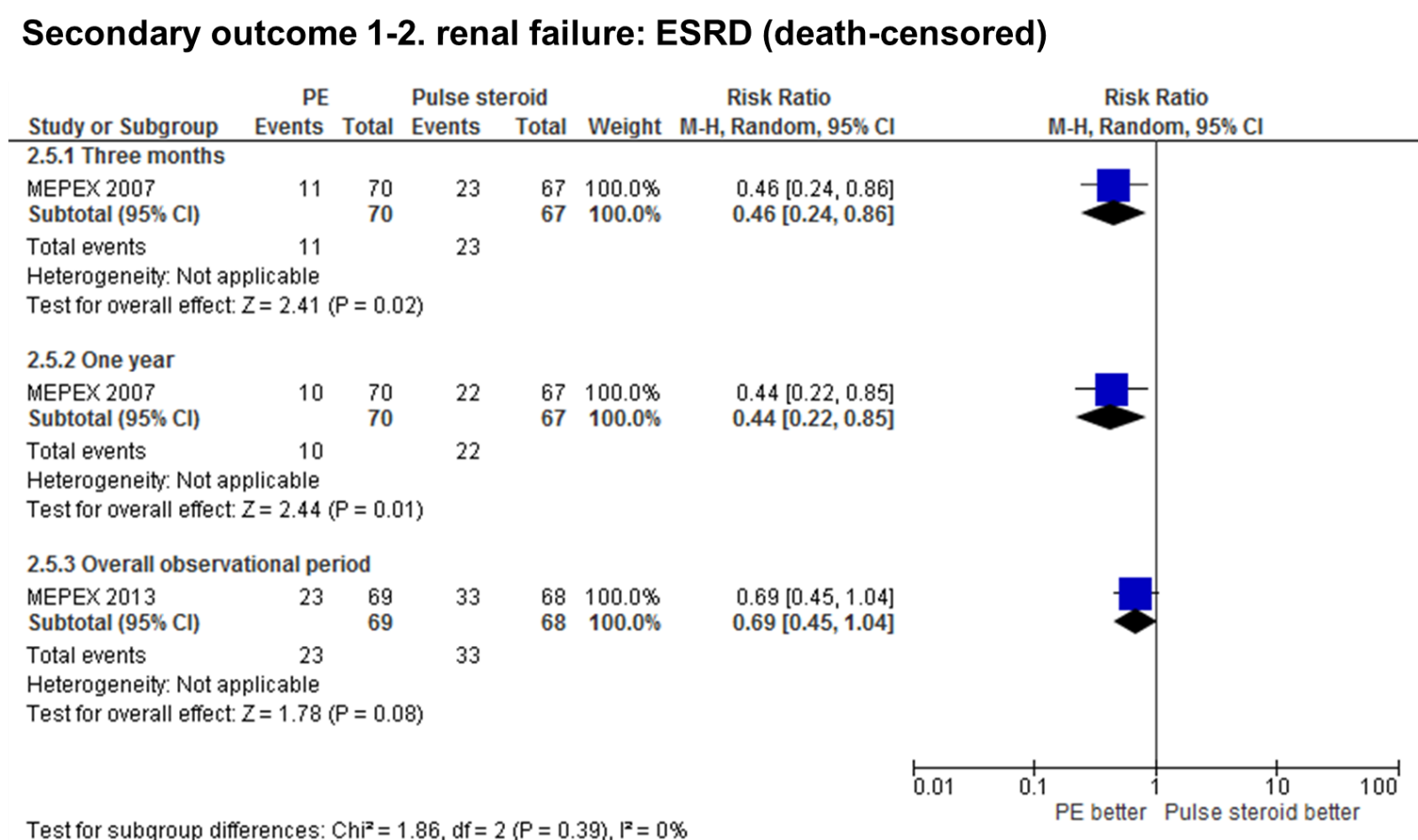
**

**Supplementary figure S14. Forest plot of end-stage renal disease (ESRD) (death-censored) in patients with plasma exchange (PE) or pulse steroid treatment.**

**
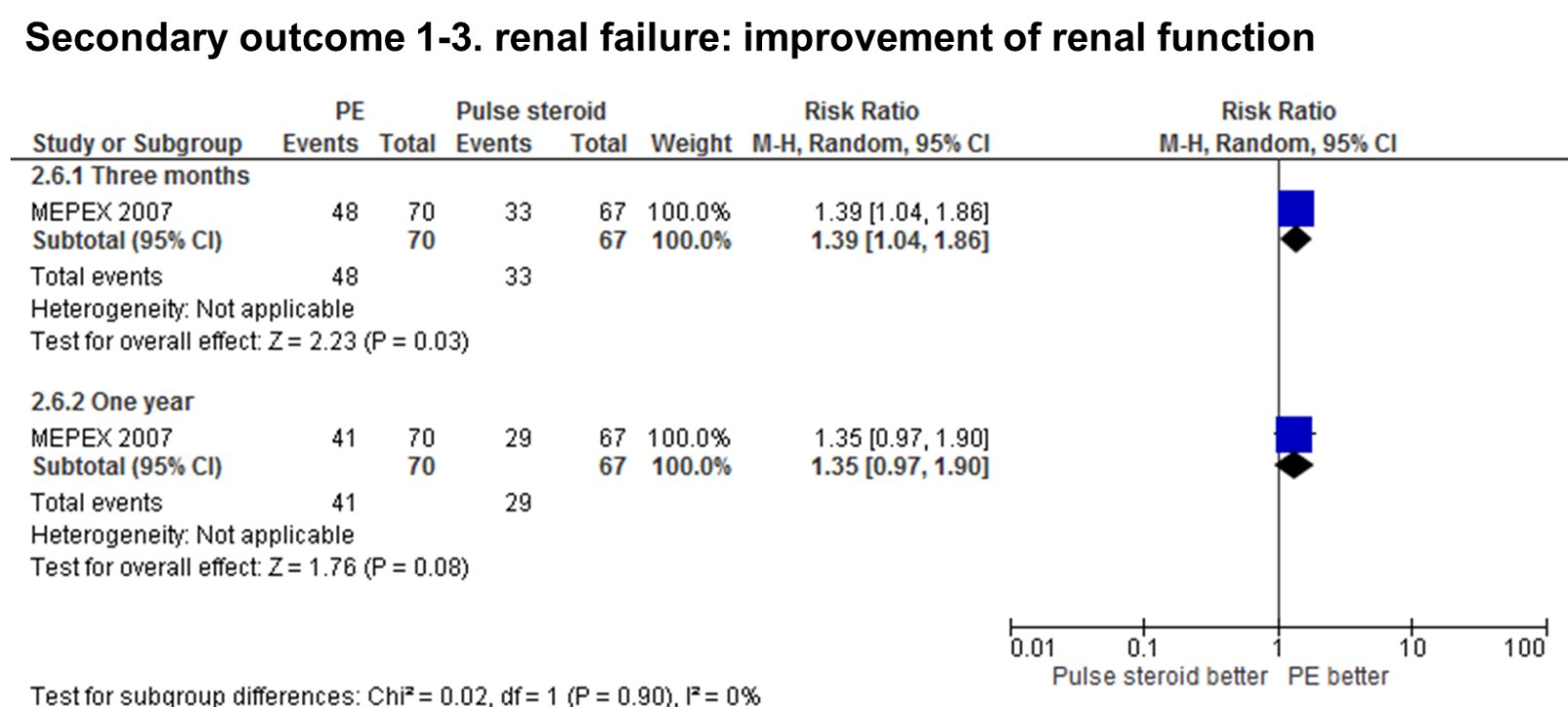
**

**Supplementary figure S15. Forest plot of improvement of renal function in patients with plasma exchange (PE) or pulse steroid treatment.**

**
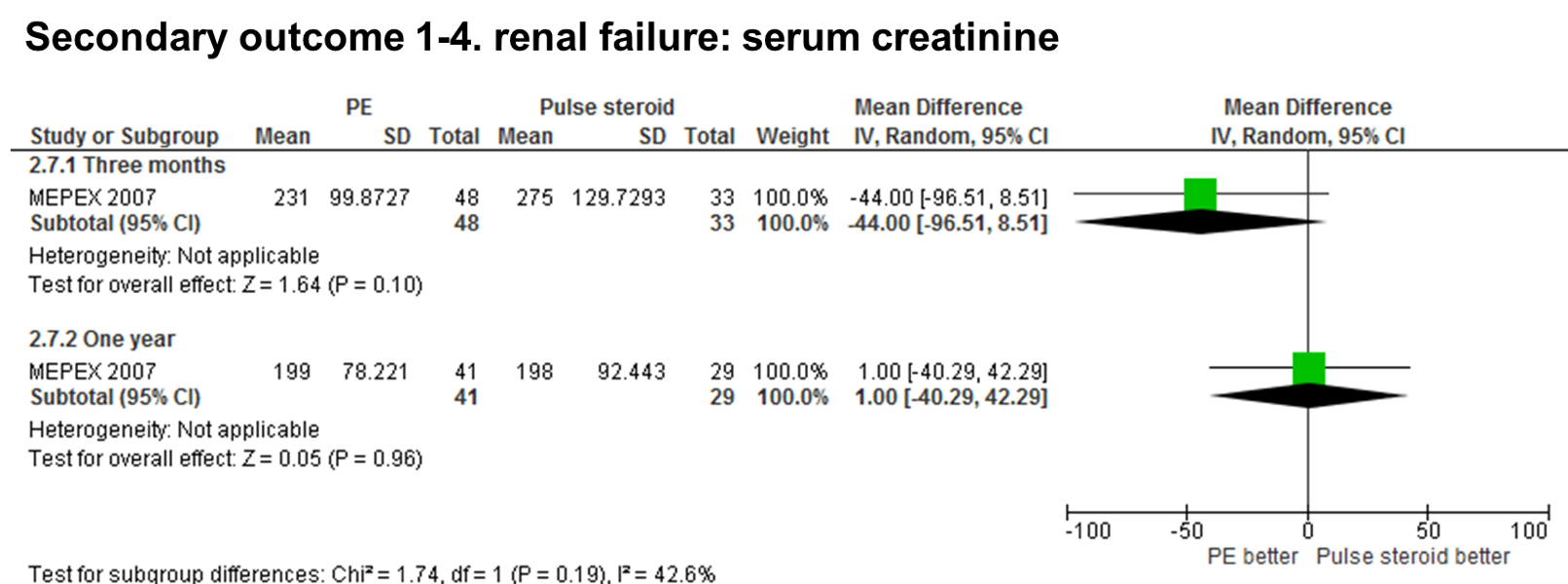
**

**Supplementary figure S16. Forest plot of serum creatinine in patients with plasma exchange (PE) or pulse steroid treatment.** Unit, µmol/L


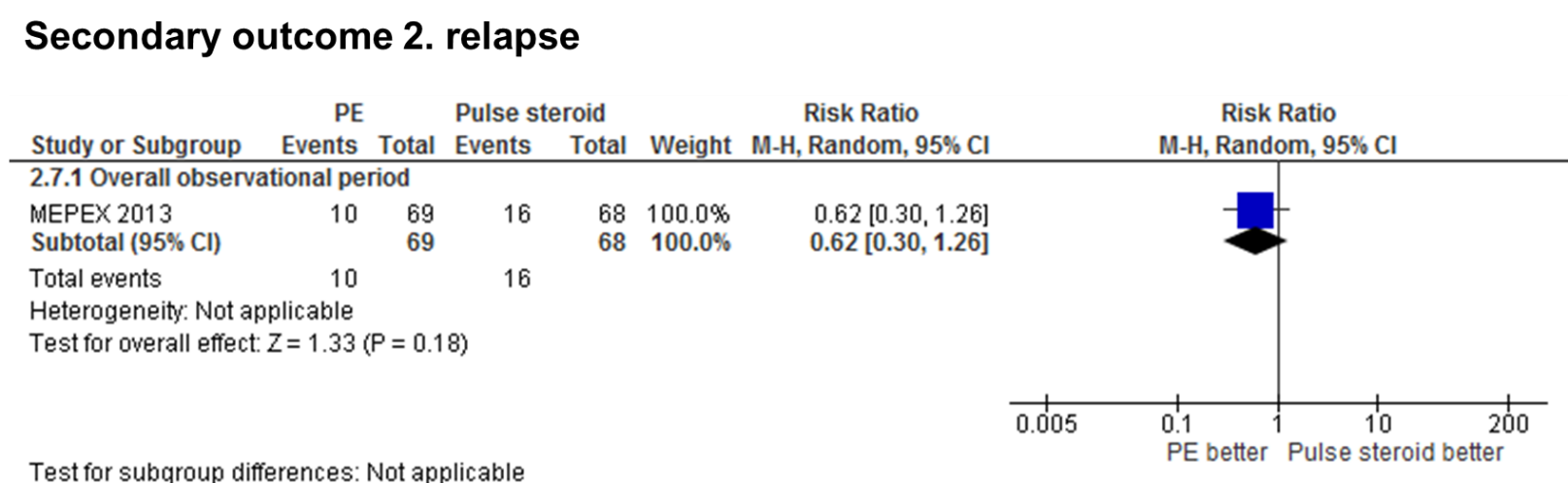


**Supplementary figure S17. Forest plot of relapse in patients with plasma exchange (PE) or pulse steroid treatment.**


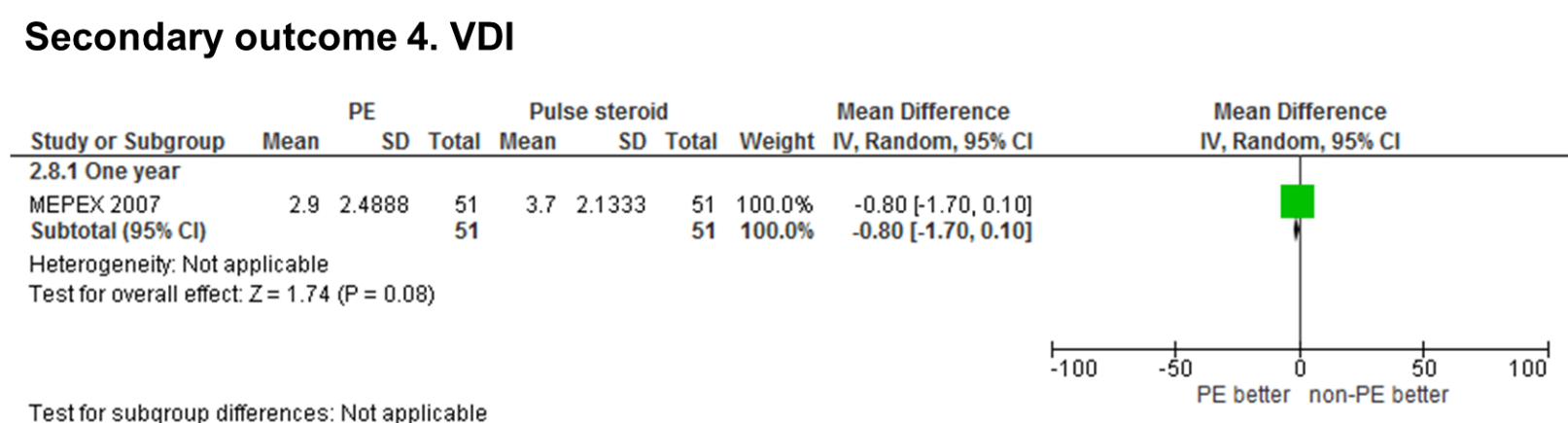


**Supplementary figure S18. Forest plot of** **vasculitis damage index (VDI) in patients with plasma exchange (PE) or pulse steroid treatment.**


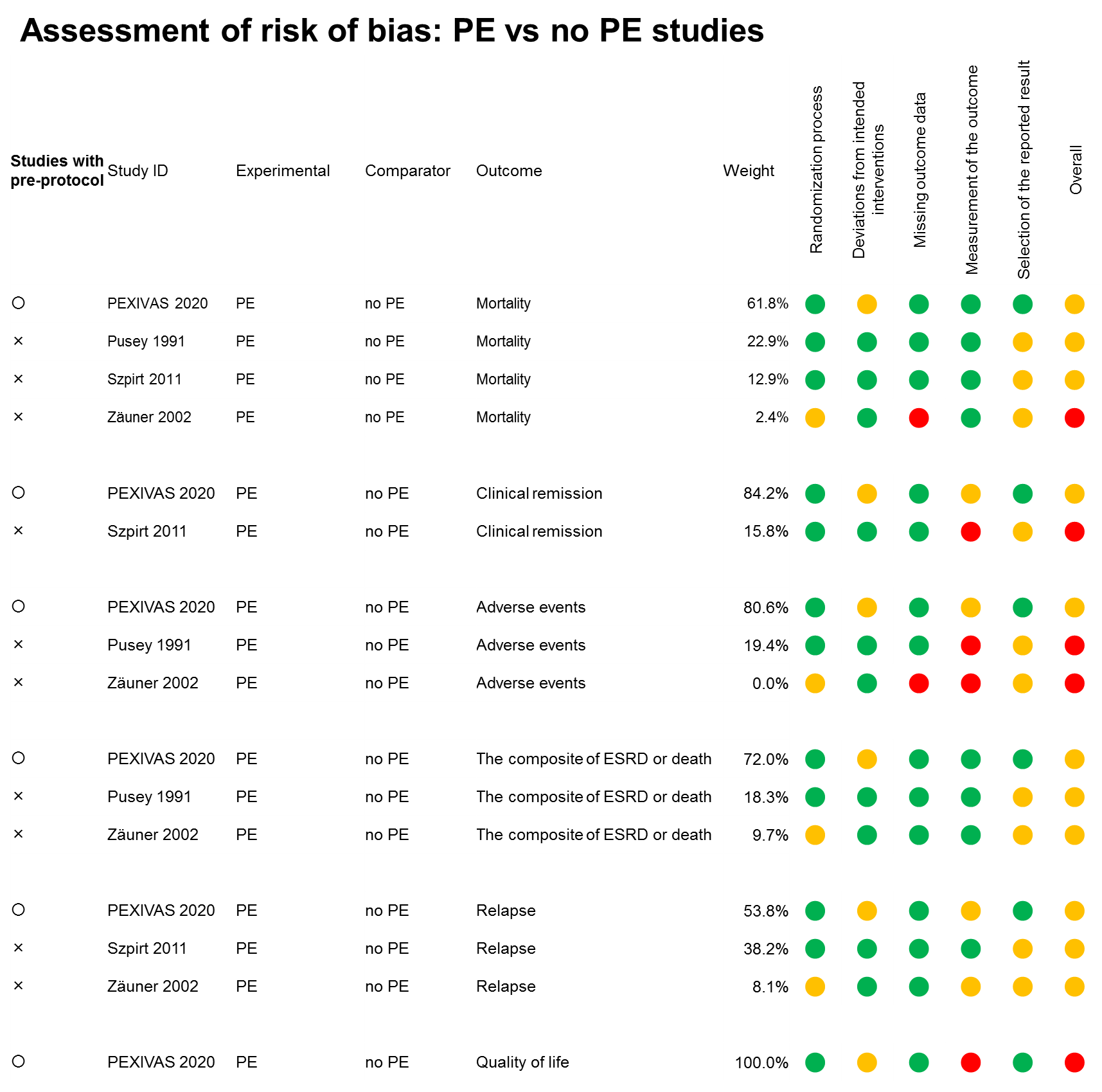


S**upplementary figure S19. Assessment of risk of bias of plasma exchange (PE) vs no PE studies, using the Cochrane 'Risk of bias' tool 2.** Green circle, low risk of bias; Yellow circle, some concerns; Red circle, high risk of bias

**
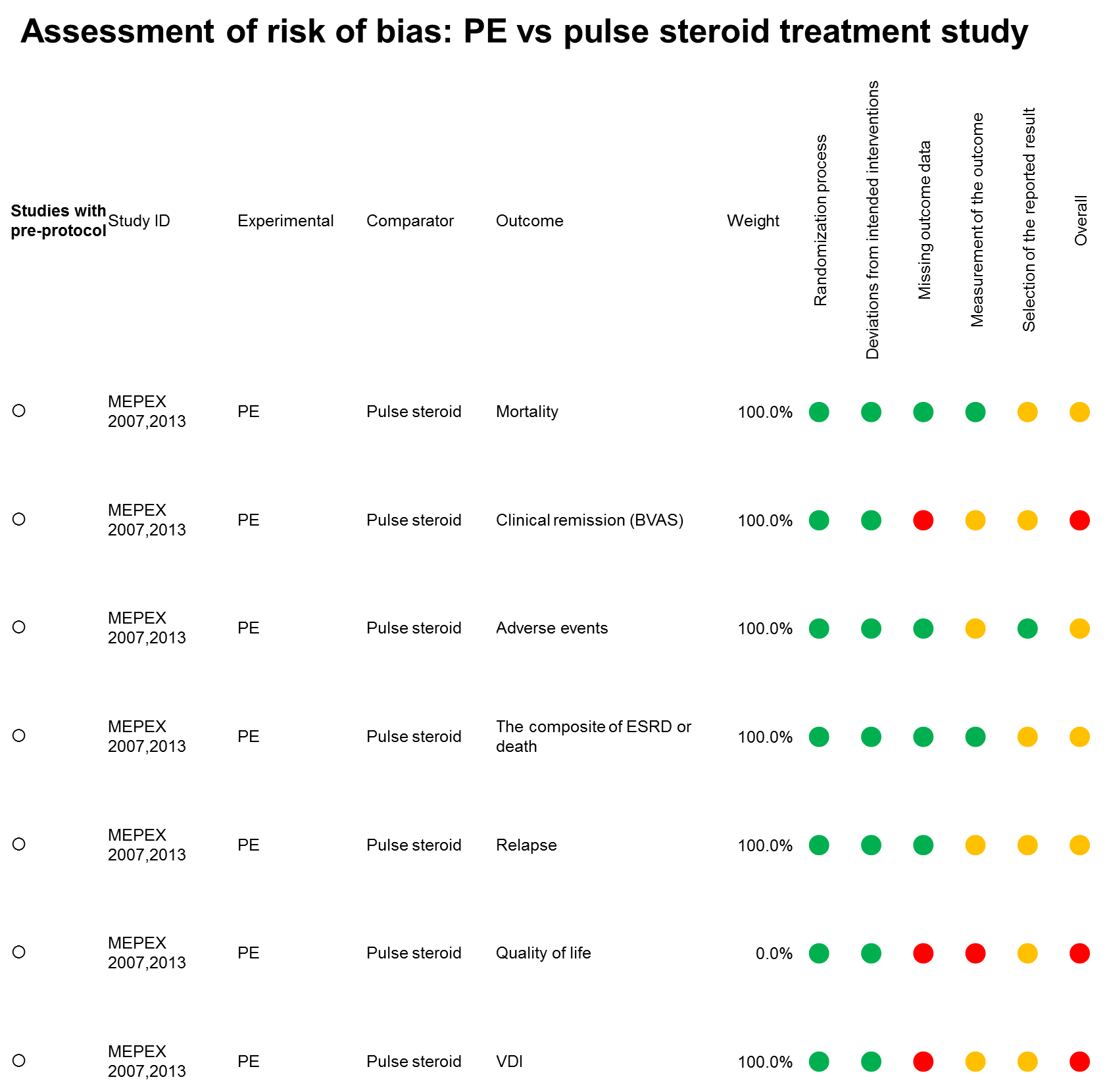
**

S**upplementary figure S20. Assessment of risk of bias of plasma exchange (PE) vs pulse steroid treatment study, using the Cochrane 'Risk of bias' tool 2.** Green circle, low risk of bias; Yellow circle, some concerns; Red circle, high risk of bias

**
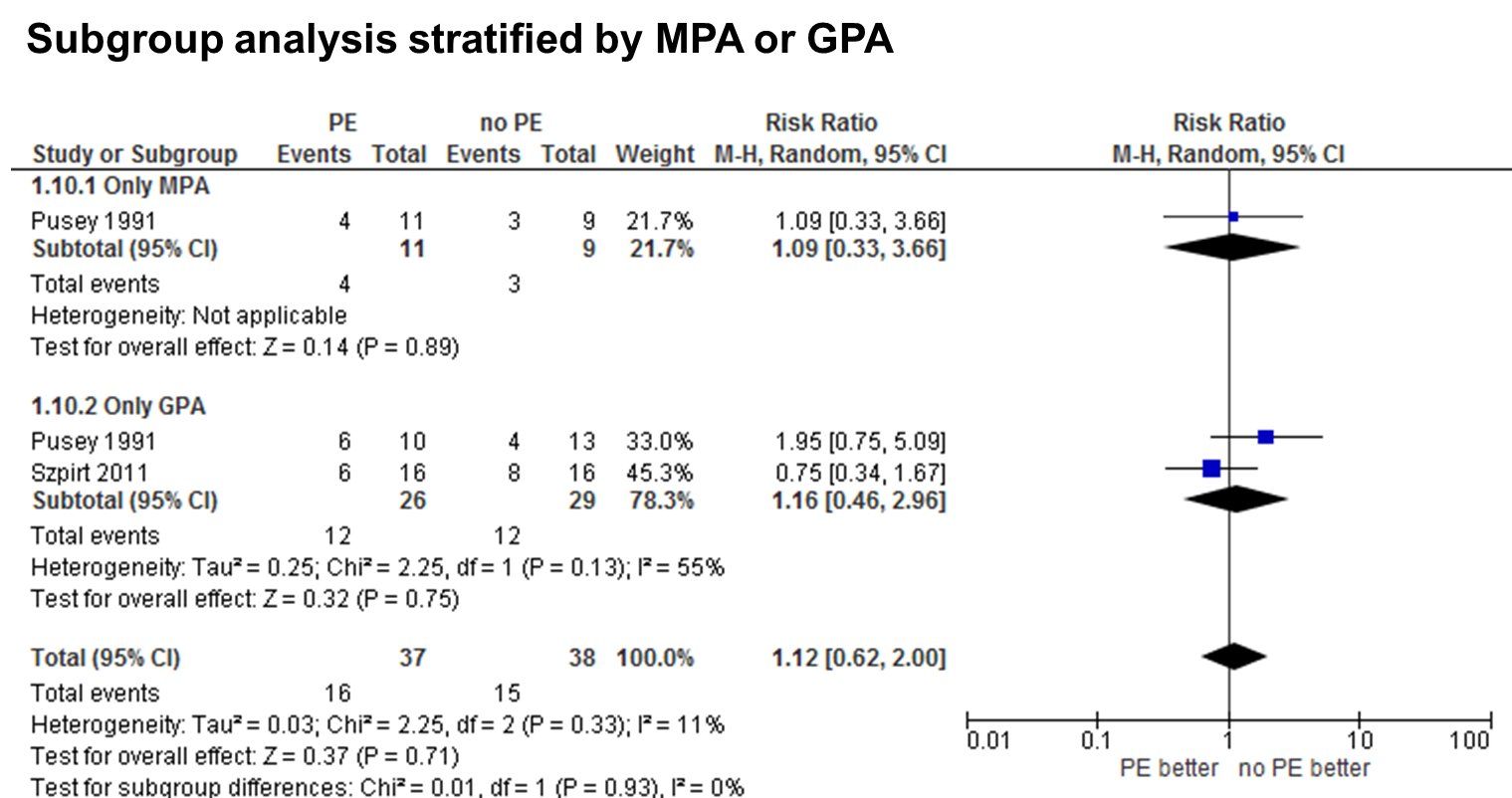
**

S**upplementary figure S21. Subgroup analysis considering mortality in patients with plasma exchange (PE) or no PE, stratified by only patients suffered from microscopic polyangiitis (MPA) or granulomatosis with polyangiitis (GPA).**

**
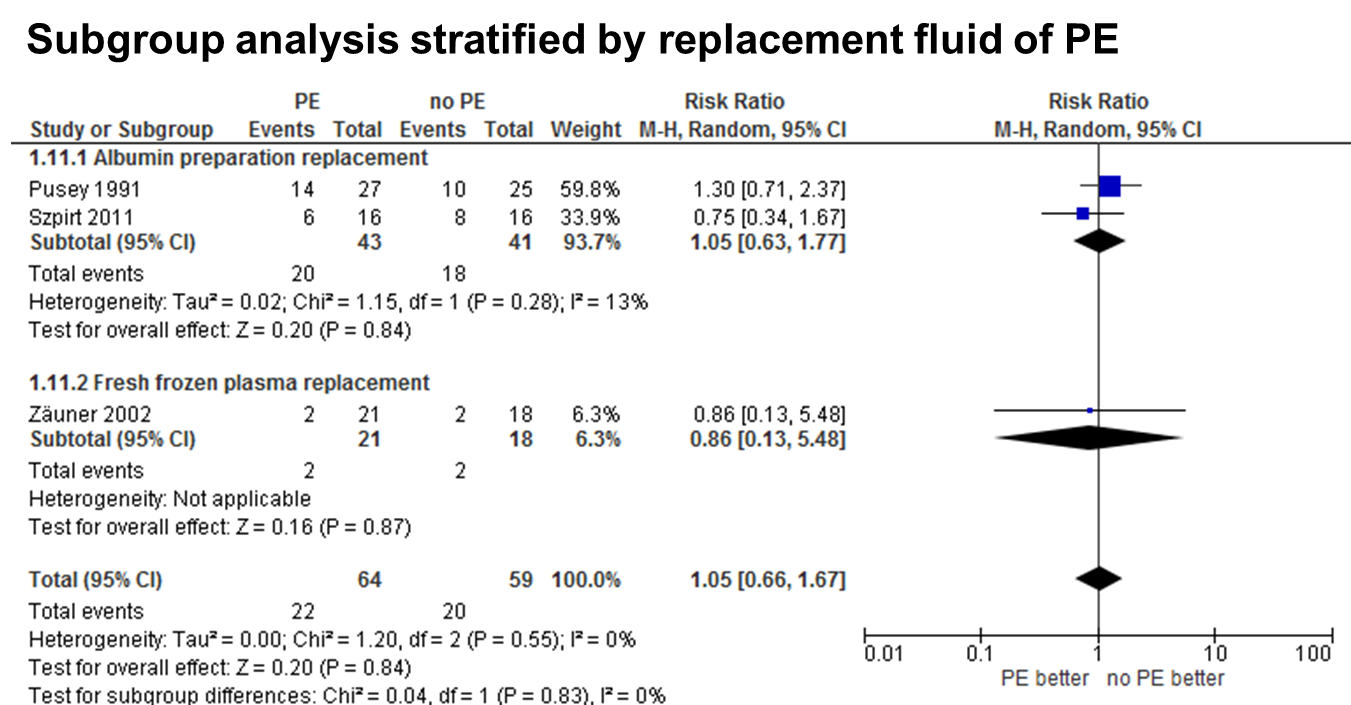
**

S**upplementary figure S22. Subgroup analysis considering mortality in patients with plasma exchange (PE) or no PE, stratified by PE replacement fluid, albumin preparation or fresh frozen plasma.**

**
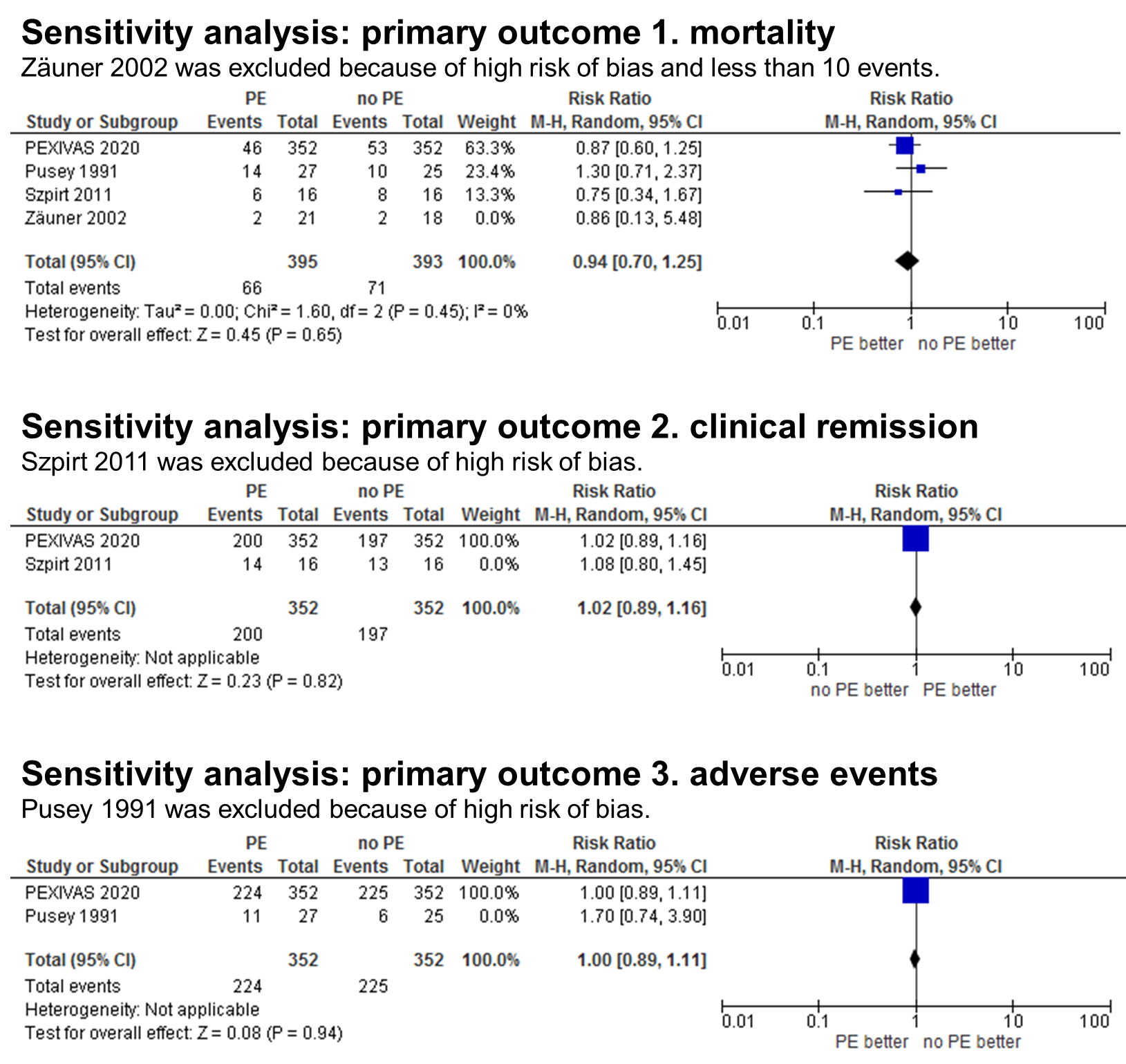
**

**Supplementary figure S23. Results of sensitivity analysis in PE vs no PE studies:** **exclusion of studies at high risk of bias or trials with ≤10 events.** Similar results were observed between the primary analysis and sensitivity analysis.

**Supplementary table S2. Sensitivity analysis comparing fixed-effect pooled estimates or 95% CIs versus random-effect pooled estimates or 95% CIs in PE vs no PE studies**

| Mortality |  |
| --- | --- |
| Random-effect model | RR 0.93 (95% CI, 0.70–1.24) |
| Fixed-effect model | RR 0.92 (95% CI, 0.68–1.23) |
|  |  |
| Clinical remission |  |
| Random-effect model | RR 1.02 (95% CI, 0.91–1.15) |
| Fixed-effect model | RR 1.02 (95% CI, 0.90–1.15) |
|  |  |
| Adverse events |  |
| Random-effect model | RR 1.10 (95% CI, 0.73–1.68) |
| Fixed-effect model | RR 1.01 (95% CI, 0.91–1.13) |

Similar results were observed between fixed-effect and random-effect models.
